# Ab Initio Prediction of Transcription Factor Targets Using Structural Knowledge

**DOI:** 10.1371/journal.pcbi.0010001

**Published:** 2005-06-24

**Authors:** Tommy Kaplan, Nir Friedman, Hanah Margalit

**Affiliations:** 1 School of Computer Science and Engineering, The Hebrew University, Jerusalem, Israel; 2 Department of Molecular Genetics and Biotechnology, Faculty of Medicine, The Hebrew University, Jerusalem, Israel; University of California at San Francisco, United States of America

## Abstract

Current approaches for identification and detection of transcription factor binding sites rely on an extensive set of known target genes. Here we describe a novel structure-based approach applicable to transcription factors with no prior binding data. Our approach combines sequence data and structural information to infer context-specific amino acid–nucleotide recognition preferences. These are used to predict binding sites for novel transcription factors from the same structural family. We demonstrate our approach on the Cys_2_His_2_ Zinc Finger protein family, and show that the learned DNA-recognition preferences are compatible with experimental results. We use these preferences to perform a genome-wide scan for direct targets of *Drosophila melanogaster* Cys_2_His_2_ transcription factors. By analyzing the predicted targets along with gene annotation and expression data we infer the function and activity of these proteins.

## Synopsis

Cells respond to dynamic changes in their environment by invoking various cellular processes, coordinated by a complex regulatory program. A main component of this program is the regulation of transcription, which is mainly accomplished by transcription factors that bind the DNA in the vicinity of genes. To better understand transcriptional regulation, advanced computational approaches are needed for linking between transcription factors and their targets. The authors describe a novel approach by which the binding site of a given transcription factor can be characterized without previous experimental binding data. This approach involves learning a set of context-specific amino acid–nucleotide recognition preferences that, when combined with the sequence and structure of the protein, can predict its specific binding preferences. Applying this approach to the Cys_2_His_2_ Zinc Finger protein family demonstrated its genome-wide potential by automatically predicting the direct targets of 29 regulators in the genome of the fruit fly *Drosophila melanogaster.* At present, with the availability of many genome sequences, there are numerous proteins annotated as transcription factors based on their sequence alone. This approach offers a promising direction for revealing the targets of these factors and for understanding their roles in the cellular network.

## Introduction

Specific binding of transcription factors to *cis*-regulatory elements is a crucial component of transcriptional regulation. Previous studies have used both experimental and computational approaches to determine the relationships between transcription factors and their targets. In particular, probabilistic models were employed to characterize the binding preferences of transcription factors, and to identify their putative sites in genomic sequences [1,2]. This approach is useful when binding data are available, but cannot be applied to proteins without extensive experimental binding studies. This difficulty is particularly emphasized in view of the genome projects, where new proteins are classified as DNA-binding according to their sequence, yet there is no information about the genes they regulate.

To address the challenge of profiling the binding sites of novel proteins, we propose a family-wise approach that builds on structural information and on the known binding sites of other proteins from the same family. We use solved protein–DNA complexes [3] to determine the exact architecture of interactions between nucleotides and amino acids at the DNA-binding domain. Although sharing the same structure, different proteins from a structural family have different binding specificities because of the presence of different residues at the DNA-binding positions. To predict their binding site motif, we need to identify the residues at these positions and understand their DNA-binding preferences.

In previous studies, we used the empirical frequencies of amino acid–nucleotide interactions [4,5] in solved complexes (from various protein families) to build a set of “DNA-recognition preferences.” This approach assumed similar DNA-binding preferences of the amino acids for all structural domains and at all binding positions. However, there are clear experimental indications that this assumption is not always valid: a particular amino acid may have different binding preferences depending on its positional context [6–8]. To estimate these context-specific DNA-recognition preferences, we need to determine the appropriate context of each residue, which may depend on its relative position and orientation with respect to the nucleotide. Then, we need to collect statistics about the DNA-binding preferences in this context. This can be achieved from an ensemble of solved protein–DNA complexes from the same family. Unfortunately, sufficient data of this type are currently unavailable.

To overcome this obstacle, we propose to estimate context-specific DNA-recognition preferences from available sequence data using statistical estimation procedures. The input of our method is a set of pairs of transcription factors and their target DNA sequences [2]. We then identify the residues and nucleotides that participate in protein–DNA interaction, and collect statistics about the DNA-binding preferences of residues under different contexts of the binding domain. These are then used to discover the binding site of other transcription factors from the same family, for which no targets are known.

**Figure 1:**
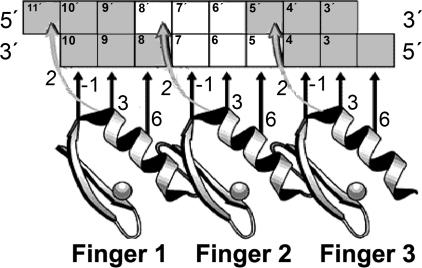
The Canonical Cys_2_His_2_ Zinc Finger DNA Binding Model Residues at positions 6, 3, 2, and −1 (relative to the beginning of the α-helix) at each finger interact with adjacent nucleotides in the DNA molecule (interactions shown with arrows). (Figure adapted from a figure by Prof. Aaron Klug, with permission.)

**Figure 2:**
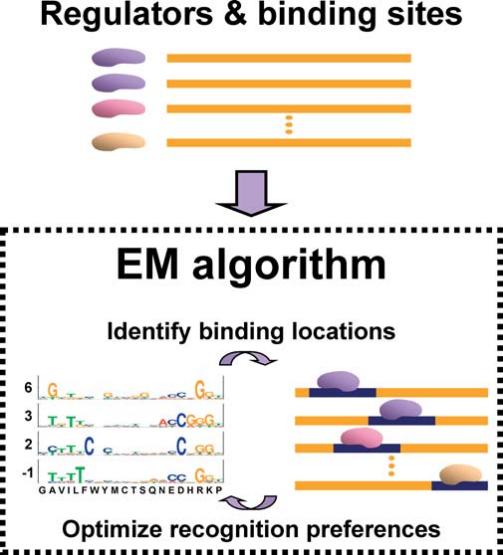
Estimating DNA-Recognition Preferences The DNA-recognition preferences are estimated from unaligned pairs of transcription factors and their DNA targets [2] (above). The EM algorithm [13] is used to simultaneously assess the exact binding positions of each protein–DNA pair (bottom right), and to estimate four sets of position-specific DNA-recognition preferences (bottom left).

**Figure 3:**
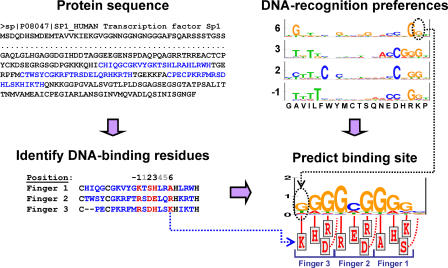
Predicting the DNA Binding Site Motifs of Novel Transcription Factors The protein's DNA-binding domains are identified using the Cys_2_His_2_ conserved pattern (top left). The residues at the key positions (6, 3, 2 and −1) of each finger (marked in red in the bottom left panel) are then assigned onto the canonical binding model (bottom right), and the sets of position-specific DNA-recognition preferences (top right panel) are used to construct a probabilistic model of the DNA binding site. For example, the lysine at the sixth position of the third finger faces the first position of the binding site (dotted blue arrow). We predict the nucleotide probabilities at this position using the appropriate recognition preferences (dotted black arrow).

We apply our approach to the Cys_2_His_2_ Zinc Finger DNA-binding family. This family is the largest known DNA-binding family in multicellular organisms [9] and has been studied extensively [10]. Members of this family bind DNA targets according to a stringent binding model [11,12], which maps the exact interactions between specific residues in the DNA-binding domain with nucleotides at the DNA site (Figure 1). We use many Zinc Finger proteins together with their native DNA targets (extracted from the TRANSFAC database [2]), and apply an iterative expectation maximization (EM) algorithm [13] to estimate position-specific DNA-recognition preferences (Figure 2). These are used in turn for predicting the DNA binding site motifs of novel proteins in the family (Figure 3), and for performing a genome-wide scan for putative targets.

## Results

### In Silico Reconstruction of DNA-Recognition Preferences

**Figure 4:**
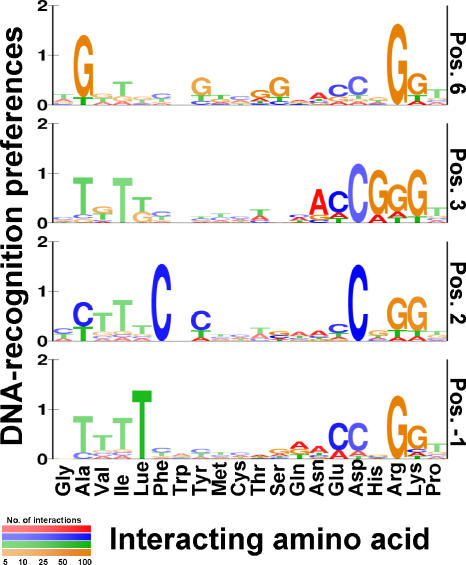
Four Sets of Position-Specific DNA-Recognition Preferences in Zinc Fingers The estimated sets of DNA-recognition preferences for the DNA-binding residues at positions 6, 3, 2, and −1 of the Zinc Finger domain are displayed as sequence logos. At each position, the associated distribution of nucleotides is displayed for each amino acid. The total height of letters represents the information content (in bits) of the position, and the relative height of each letter represents its probability. Color intensity indicates the level of confidence for a given amino acid at a certain position (where paler colors indicate lower confidence due to low occurrences of the amino acid at the specific position in the training data) (see Tables S1 and S2 for full data). Some of the DNA binding preferences are general, regardless of the residue's position within the zinc finger (e.g., lysine's tendency to bind guanine), while others are position-dependent (e.g., the tendency of phenylalanine to bind cytosine only when in position 2).

In order to estimate the context-specific DNA-recognition preferences of the Cys_2_His_2_ Zinc Finger DNA-binding family we used the canonical binding model learned from the solved protein–DNA complex of Egr-1 [11,12]. According to this model, the binding specificity of each Zinc Finger domain is determined by residues at four key positions (see Figure 1). We aimed to learn a different set of DNA-recognition preferences for each of the four key positions. These sets should express the probability of every amino acid to interact with each nucleotide. Since the number of solved protein–DNA complexes is insufficient to estimate these preferences directly, we resorted to sequence data of proteins and their DNA targets. We extracted 455 protein–DNA pairs from the TRANSFAC 7.3 database [2] (see Materials and Methods). Unfortunately, the exact binding locations of these DNA targets are not pinpointed, and thus we employed statistical tools to infer them (see Figure 2; Materials and Methods). We then used the protein–DNA binding model to identify the interacting residues and nucleotides, and collect statistics on their binding preferences (see Materials and Methods). Based on these we estimated four sets of DNA-recognition preferences (Figure 4; Tables S1 and S2), showing both context-independent preferences (such as the preference of lysine for guanine) and context-dependent ones (e.g., the preference of aspartic acid for cytosine). Table S3 shows the 10%–90% confidence intervals of the estimated probabilities.

### Learned Recognition Preferences Are Consistent with Experimental Results

We evaluated the four reconstructed sets of DNA-recognition preferences by comparing them with experimental data. First, we compared the derived preferences with qualitative preferences based on phage-display experiments [10] and found the two to be consistent (data not shown). Second, we predicted binding site models for Egr-1 variants for which experimental binding data were available [14], using their sequences and our estimated preferences. These models were used to score the binding of Egr-1 variants to a set of DNA targets that were tested in the experimental study. We found that our predictions were highly correlated with the experimentally measured binding affinities [14] (Table S4).

**Figure 5:**
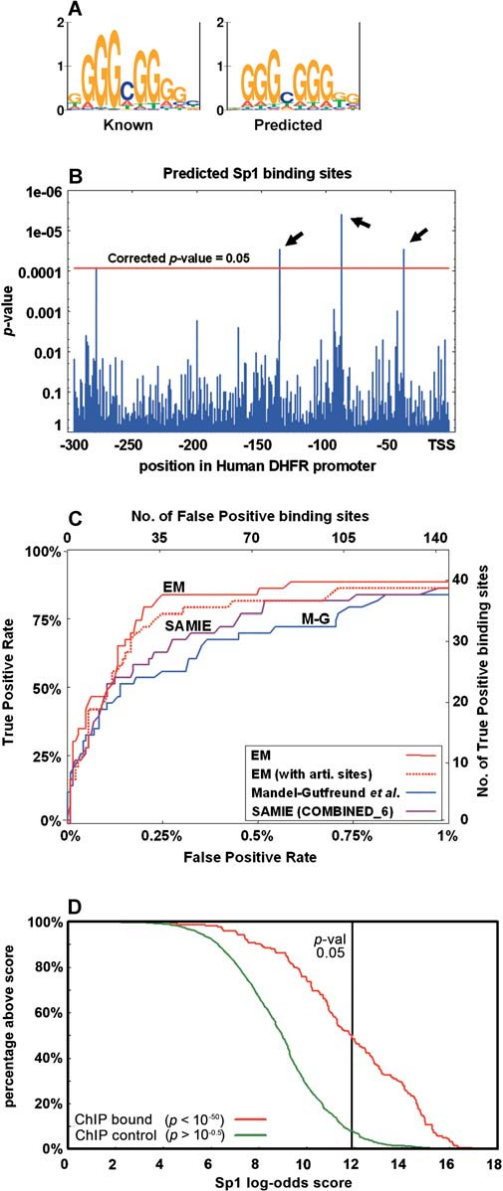
Validation of DNA-Recognition Preferences (A) The predicted binding site model of human Sp1 protein is compared to its known site (matrix V$SP1_Q6 from TRANSFAC [2], based on 108 aligned binding sites). To prevent bias by known Sp1 sites in our training data, the set of DNA-recognition preferences was estimated from the TRANSFAC data after removing all Sp1 sites. (B) Scanning the 300-bp-long promoter of human dihydrofolate reductase (DHFR) by the predicted Sp1 binding model. The *p*-value of each potential binding site is shown (*y*-axis). Four positions achieved a significant *p*-value (higher than the horizontal red line), out of which three are known Sp1 binding sites [41] (arrows). (C) A summary of in silico binding experiments for 21 pairs of Zinc Finger transcription factors and their target promoters. Shown is the tradeoff between false positive rate (*x*-axis) and true positive rate (*y*-axis) as the significance threshold for putative binding sites is changed. For every threshold point, our set of recognition preferences (EM) achieves better accuracy than the preferences of Mandel-Gutfreund et al. [5] (M-G) and Benos et al. [15] (SAMIE). Interestingly, when the DNA-recognition preferences were estimated from training data expanded to include TRANSFAC's artificial sequences, inferior results were obtained (dotted red line). (D) Cumulative distribution of Sp1 scores among the sequences of targets/non-targets of unbiased chromatin immunoprecipitation scans of human Chromosomes 21 and 22 [16]. The predicted Sp1 motif appears in 45% of the experimentally bound sequences but in only 5% of the control sequences.

Next, we evaluated the ability of the estimated recognition preferences to identify binding sites within genomic sequences. We compiled a dataset of binding sites of ten Cys_2_His_2_ transcription factors. These involved 43 experimentally verified binding sites within natural genomic promoter sequences with a total length of 14,534 bp (Table S5). Using the recognition preferences, we predicted the binding site models of the ten transcription factors and used them to scan the respective promoter regions for putative binding sites (Figure 5A and 5B; see Materials and Methods). To prevent bias by known sites in our training data, we applied a “leave protein out” cross-validation analysis, and predicted the DNA binding model of a protein using DNA-recognition preferences that were learned from a reduced dataset, from which all its binding sites were removed. Our method marked 30 locations as putative binding sites, out of which 21 matched experimental knowledge (sensitivity of 49% and specificity of 70%, *p* < 10^−48^; see Table S6).

Benos et al. [15] proposed a method (SAMIE) to estimate Cys_2_His_2_ Zinc Finger position-specific binding preferences from in vitro SELEX binding experiments. We compared the predictions of the known binding sites within promoter regions provided by our position-specific recognition preferences to those of Benos et al. [15] and of Mandel-Gutfreund et al. [5] (Figure 5C; Table S7). These results suggest that predictions based on our recognition preferences out-perform the predictions based on the other methods.

To further evaluate our predictions, we used the binding locations of Sp1 along human Chromosomes 21 and 22, as mapped by genome-wide chromatin immunoprecipitation [16]. We compiled two datasets of 1-kb-long sequences: one dataset included sequences that exhibited highly significant binding, and the other dataset included sequences that showed no binding at all (to be used as a control; see Materials and Methods). We used the DNA-recognition preferences to predict a binding site model for Sp1, and scanned the genomic sequences with it. We identified Sp1 binding sites in 45% of the experimentally bound sequences, and in only 5% of the control sequences (Figure 5D).

### Ab Initio Genome-Wide Prediction of Transcription Factor Binding Sites

In the past few years many genomes were solved, yielding sequences of thousands of putative transcription factors. However, only little is currently known about the binding specificities of these factors and about their target genes. To address this problem, we applied our predictive scheme to the *Drosophila melanogaster* genome in a fully automated manner. We first scanned the sequences of 16,201 putative gene products and identified 29 canonical Cys_2_His_2_ Zinc Finger transcription factors with three or four fingers (see Materials and Methods). We then used their sequences and the estimated DNA-recognition preferences to compile a binding site model for each transcription factor, as in Figure 3 (see Figure S1 and Table S8 for detailed models). Finally, we used these binding site models to scan the upstream promoter regions of 15,665 *D. melanogaster* genes. Multiple putative direct targets were predicted for each Zinc Finger, as detailed at http://compbio.cs.huji.ac.il/Zinc. The number of putative direct target genes for each transcription factor and the overlap between targets of different factors are shown in Figures S2 and S3. Interestingly, several Zinc Fingers have similar residues at the DNA-binding positions, and are therefore predicted to bind similar sites and to have mutual predicted targets (see Figures S1 and S3). In *D. melanogaster,* this phenomenon has been reported for at least some transcription factors (e.g., Sp1 and Btd) [17].

**Figure 6:**
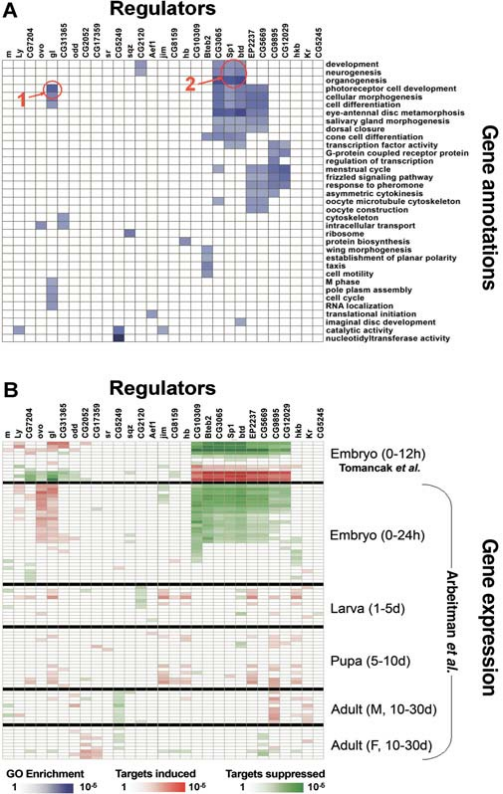
Inferring the Function and Activity of Zinc Finger Transcription Factors in *D. melanogaster* (A) Similar gene annotation enrichment among the putative target sets of 29 transcription factors in *D. melanogaster*. Blue cells correspond to significant overabundance of a GO term (row) among the predicted targets of a protein (column), using a hyper-geometric test. The binding sites of most factors show enrichment in at least one GO term. For some of the regulators, the enriched GO terms match prior biological knowledge. For example, the putative targets of Glass (gl) were found to be enriched with terms related to photoreceptor cell development (red circle 1). Similarly, the putative targets of Buttonhead (btd) and Sp1 were enriched with developmental terms such as neurogenesis, development, and organogenesis (red circle 2). Closely related GO annotations are not shown; see Figure S4 for full results. (B) Deducing the activity of the 29 transcription factors using gene expression patterns. Expression data from early (0–12 h) embryogenesis [20] and data from the entire *Drosophila* life cycle [21] are used to test whether the putative direct targets of a regulator are expressed differently than the rest of the genes in a given experiment. Red cells correspond to significant enrichment of overexpressed targets using a Kolmogorov-Smirnov test, while green cells correspond to enrichment of underexpressed targets. For most of the regulators the analysis resulted in at least one significant embryogenesis experiment, suggesting an active role in early developmental stages (above). Similar results were obtained using the full life cycle gene expression data (below).

To infer the function of the 29 transcription factors, we employed the functional annotations of their predicted target genes (based on the Gene Ontology [GO] terms [18]). The target sets of most transcription factors (21 out of 29) were found to be significantly enriched with at least one GO term (Figure 6A). For some of the transcription factors, the enriched GO terms match prior biological knowledge. For example, the putative targets of Glass were found to be enriched with terms related to photoreceptor cell development, consistent with previous studies that linked the Glass transcription factor with eye photoreceptor development [19]. Similarly, the putative targets of Btd and Sp1 were enriched with developmental terms, such as neurogenesis, development, and organogenesis. Indeed these regulators are known to play essential roles in mechanosensory development [17]. Furthermore, our analysis suggests possible functions for unknown proteins, as well as new annotations for some of the already known regulators (see Figure S4 for complete results).

We further evaluated the function and activity of the 29 transcription factors based on the mRNA expression profiles of their target genes (Figure 6B). We used expression data from early embryogenesis [20], as well as data from the entire life cycle of *D. melanogaster* [21]. In each experiment and for each transcription factor, we tested whether its putative targets showed similarity in their expression patterns and differed from the rest of the genes (see Materials and Methods). Such coherent expression supports the suggested relationship between the factor and the genes it is predicted to regulate. Out of the 29 transcription factors we examined, 21 showed such significant associations in at least one embryogenesis experiment, suggesting their active roles throughout early developmental stages (Figure 6B). These transcription factors include many known developmental regulators that are active during embryonic development (e.g., Btd, Sp1, Glass, Odd-skipped, and Stripe) [18,22], as well as other proteins, whose function is currently unknown. Similar results were obtained using the full life cycle gene expression data [21], mapping putative time points at which each regulator is predicted to be active (Figure 6B).

Note that the expression profiles are based on whole embryos, and therefore ignore spatially differential expression patterns. Thus, the correct function of some tissue-specific Zinc Finger proteins may be obscured in these data. Additional insight may be gained by focusing on expression data in homogeneous regions. Specifically, Butler et al. [23] compared gene expression in two homogeneous parts of the *Drosophila* imaginal wing disc—the body wall and the hinge-wing pouch. In our analysis we used the ratios between the expression levels in the two regions, and examined putative targets for enrichment in one of the regions. We then inferred the regulatory role of a transcription factor (activator or repressor) using its own expression pattern. For example, the putative targets of Stripe show higher expression levels in the body wall than the rest of the genes (enrichment *p*-value ≤ 0.0002). Stripe itself is enriched more than 9-fold in the body wall, relative to the wing-hinge region. This suggests that Stripe functions mainly in the body-wall region, where it activates its target genes. Indeed, this is consistent with the known role of Stripe as an activator of epidermal muscle attachment genes [24]. Using the same reasoning, we inferred the regulatory roles of four additional *D. melanogaster* transcription factors within the imaginal wing disc, three of which were previously uncharacterized (Table 1).

**Table 1:**
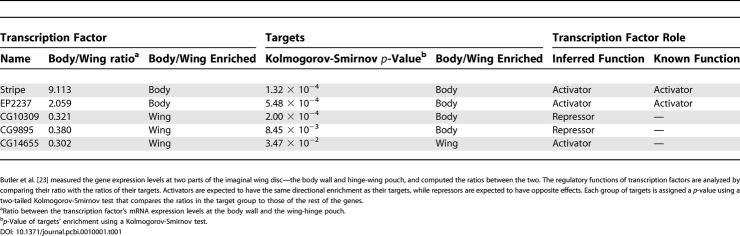
Analysis of Differential Expression in *D. melanogaster* Imaginal Wing Disc

Butler et al*.* [23] measured the gene expression levels at two parts of the imaginal wing disc—the body wall and hinge-wing pouch, and computed the ratios between the two. The regulatory functions of transcription factors are analyzed by comparing their ratio with the ratios of their targets. Activators are expected to have the same directional enrichment as their targets, while repressors are expected to have opposite effects. Each group of targets is assigned a *p*-value using a two-tailed Kolmogorov-Smirnov test that compares the ratios in the target group to those of the rest of the genes.

^a^Ratio between the transcription factor's mRNA expression levels at the body wall and the wing-hinge pouch.

^b^ *p*-Value of targets' enrichment using a Kolmogorov-Smirnov test.

## Discussion

In this paper we propose a general framework for predicting the DNA binding site sequence of novel transcription factors from known families. Our framework combines structural information about a specific DNA-binding domain with examples of binding sites for proteins in the family. We apply a statistical estimation algorithm to the canonical Cys_2_His_2_ Zinc Finger DNA-binding family, and derive a set of DNA-recognition preferences for each residue at each interacting position in the Zinc Finger DNA-binding domain.

We apply these preferences and predict the binding site models of novel proteins from the same family. Finally, we use the predicted models in genome-wide scans and identify the proteins' putative direct target genes.

Structure-based approaches for prediction of transcription factor binding sites have recently gained much interest [5,8,15,25–29]. Most of the current structural approaches define a binding model based on solved protein–DNA complexes, and attempt to identify DNA subsequences that best fit the amino acids that are determined as interacting with the DNA. Previous studies [4,8] used ensembles of solved protein–DNA complexes (from all DNA-binding domains) to extract general parameters for amino acid–base recognition. Some studies used only the counts of amino acid–nucleotide pairs to derive these parameters [4], whereas others also considered the spatial arrangements [8]. However, for fine grained definition of such potentials, a much larger set of solved protein–DNA complexes is needed than is currently available. An alternative approach to estimate DNA-recognition preferences is to extract them separately for each DNA-binding domain. However, here again, the data of solved complexes are insufficient to allow such derivation.

In a recent study, Benos et al. [15] assigned position-specific DNA-recognition preferences for the Cys_2_His_2_ Zinc Finger family. The model they used is similar to ours, with two significant differences. First, they relied on data from in vitro selection assays, such as SELEX and phage display, to train their recognition preferences. Second, their assays screened artificial sequences, both artificial proteins and artificial DNA targets. In contrast, we rely on previously published information of natural binding sites. Our approach does not require specialized experiments, and more importantly, it captures the specificity of natural proteins to DNA sequences. As we showed, our preferences are consistent with independent experimental results [6,7,10] and are superior to such preferences derived by the other computational approaches [5,15]. In addition, previous studies showed that there are discrepancies between SELEX-derived motifs and those derived from natural binding sites [30,31]. Indeed, our method yielded inferior predictions when information on artificial binding sequences was included in our training data. Figure 4C shows that our set of recognition preferences is superior to previous models in identifying genomic binding sites. When comparing the predictions by the various recognition preferences to measured affinities of DNA artificial sequences [14], we report results similar to those of Benos et al. (see Table S4).

### Analysis of the Estimated DNA-Recognition Preferences

Analysis of the estimated recognition preferences suggests that the protein–DNA recognition code is not deterministic, but rather spans a range of preferences. Moreover, our analyses show that a residue may have different nucleotide preferences depending on its context. For some amino acids, the qualitative preferences remain the same across various positions, while the quantitative preferences vary (e.g., arginine; see Figure 4). The DNA-binding preferences of other residues change across various positions. For example, histidine at position 3 tends to interact with guanine, while it shows no preference to any nucleotide at all other positions. Another example is the tendency of alanine at position 6 to face guanine. This preference, which was revealed automatically by our analysis, is not consistent with the chemical nature of alanine's side chain nor with general examinations of amino acid–nucleotide interactions [5,8]. We suspect that it is affected by the large number of Sp1 targets in our dataset. This potential interaction was implied before in Sp1 binding sites [32] and may reflect an interaction between the residue at position 2 with the complementary cytosine.

### The Protein–DNA Binding Model

In this work, we use a binding model that is based on solved protein–DNA complexes. The model presents a rigid and simplistic representation of the amino acid–base interactions at the Zinc Finger domains. Only some of the Zinc Finger domains (termed “canonical” in this work) use this model for binding, while others maintain more complex interactions. As our results show, by using this model, we manage to recover most of the DNA-binding specificities of amino acids, and use them to predict the binding site models of novel proteins. We believe that this model offers a fair tradeoff between complexity (and number of parameters) and accuracy.

### Inter-Position Dependencies in the Binding Site

The Cys_2_His_2_ binding model inherently assumes that all positions within the binding site are independent of each other. This assumption is used in most computational approaches that model binding sites. Two recent papers [33,34] discuss this issue in the context of the Cys_2_His_2_ Zinc Finger domain. Their analyses of binding affinity measurements suggest that weak dependencies do exist among some positions of the binding sites of Egr-1. Nonetheless, a reasonable approximation of the binding specificities is obtained even when ignoring these dependencies. In another recent study [35], we evaluated probabilistic models that are capable of capturing inter-position dependencies within binding sites. Our results show that dependencies can be found in the binding sites of many proteins from various DNA-binding domains (especially from the helix-turn-helix and the homeo domains). However, our results also suggest that such models of dependencies do not lead to significant improvements in modeling the binding sites of Zinc Finger proteins. Thus, we believe that the Cys_2_His_2_ binding model we use here is indeed a reasonable approximation of the actual binding.

### Genome-Wide Predictions of Binding Sites and Target Genes

In the current era there is a growing gap between the number of known protein sequences and the number of experimentally verified binding sites. To better understand regulatory mechanisms in newly solved genomes, it is crucial to identify the direct target genes of novel DNA-binding proteins. Our method opens the way for such genome-wide assays. Here we apply it to the Cys_2_His_2_ Zinc Finger DNA-binding family. By predicting the binding site models of regulatory proteins, one can classify genes into those that contain significant binding sites at their regulatory promoter regions (hence, putative target genes) and those that do not. As we showed, our approach can scale up to such genome-wide scans and successfully predict the target genes of many novel Zinc Finger proteins in higher eukaryotes. Furthermore, by integrating data from external sources, such as gene expression and GO annotations, it is possible to infer the cellular function and activity of these novel proteins.

### Applications to Other DNA-Binding Domains

Theoretically, our approach can be extended to handle other structural families, such as the basic leucine zipper, the homeodomain, and the basic helix-loop-helix, for which enough binding data already exist (1,191, 505, and 201 binding sites per family, respectively). This extension requires that the various proteins in the family show a common DNA binding model, which can be used further for other family members. For such families, our approach should suffice. For other families, where the binding models are more complex and flexible (including other Zinc Finger domains, such as CCCC, CCHC, or even the non-canonical Cys_2_His_2_), more advanced models and learning techniques will be needed. In spite of these possible difficulties, we believe that structural approaches, such as the one we show here, open promising directions, leading to successful predictions of binding site models and, following that, to accurate identification of the target genes of novel proteins, even on genome-wide scales. Eventually, such approaches will be utilized to reconstruct larger and larger portions of the transcriptional regulatory networks that control the living cell.

## Materials and Methods

### 

#### Sequences of Zinc Finger proteins and their binding sites.

We trained a profile hidden Markov model [36] on 31 experimentally determined canonical domains [37], and used it to classify the remaining Cys_2_His_2_ Zinc Finger domains in TRANSFAC [2] as canonical or non-canonical. From these, we selected proteins with two to four properly spaced canonical fingers. This resulted in 61 canonical Cys_2_His_2_ Zinc Finger proteins, and 455 protein–binding site pairs. We used these pairs as our training data in subsequent steps. The total number of fingers in this dataset was 1,320, and the total length of all binding sites was 9,761 bp (average length of 21 bp per site).

#### Identification of DNA-binding residues.

The interacting residues in each finger are located at positions 6, 3, 2, and −1 relative to the beginning of the α-helix (see Figure 1). We identify these positions using their relative positioning in the Cys_2_His_2_ conserved pattern: CX(2–4)CX(11–13)HX(3–5)H. Although, theoretically there can be 20^4^ different combinations of amino acids at the interacting positions, we found only 80 different combinations among the 1,320 fingers in our database. Figures S5 and S6 show the abundance of amino acids at the different DNA-binding positions.

#### The probabilistic model.

We describe the binding preferences of a protein using a probabilistic model. For a canonical Egr-1-like Zinc Finger protein, we denote by *A* = {*A_i,p_* : *i* = {1,…, *k*}, *p* ∈ {−1,2,3,6}} the set of interacting residues in the different four positions of the *k *fingers (ordered from the N- to the C-terminus). Let *N*_1_,…, *N_L_* be a target DNA sequence. The conditional probability of an interaction with a DNA subsequence, starting from the *j*th position in the DNA is


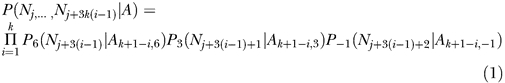


where *P_p_*(*N*|*A*) is the conditional probability of nucleotide *N* given amino acid *A* at position* p*. These probabilities are the parameters of the model. For each of the four interacting positions there is a matrix of the conditional probabilities of the four nucleotides given all 20 residues. We call these matrices the DNA-recognition preferences.

The model, as described above, does not account for the interactions by the amino acid in position 2 in each finger. According to the canonical binding model (see Figure 1), the amino acid at position 2 interacts with the nucleotide that is complementary to the nucleotide interacting with position 6 of the previous finger. Thus, when we have a base pair interacting with two amino acids, we replace the term *P*_6_(*N_j_*_+3(*i*−1)_|*A_k_*_+1−*i*,6_) with the term


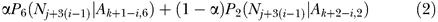


for *i* > 1, where α is a weighting coefficient that depends on the number of examples we have seen while estimating the recognition preferences at each position. Moreover, we add the term *P*_2_(*N_j_*_+3(*i*−1)_|*A_k_*_+2−*i*,2_), for *i* = *k* + 1, to capture the last nucleotide, which is in interaction with position 2 of the first finger.

#### Estimating DNA-recognition preferences.

We searched for the DNA-recognition preferences that maximized the likelihood of the DNA sites given the binding proteins. The DNA sequences in our database were reported as containing the binding sites [2], yet the exact binding locations were not pinpointed. Thus, we simultaneously identified the exact binding locations and maximum likelihood recognition preferences using the iterative EM algorithm [13]. Starting with an initial choice of DNA-recognition preferences (possible choices are discussed below), the algorithm proceeds iteratively, by carrying out two steps. In the E-step, the expected posterior probability of binding locations is computed for every protein–DNA pair. This is done using the current sets of preferences. In the M-step, the DNA-recognition preferences are updated to maximize the likelihood of the current binding positions for all protein–DNA pairs based on the distribution of possible binding locations computed in the E-step.

Each iteration of these two steps increases the likelihood of the data until reaching a convergence point [13]. Although the EM algorithm is proved to converge, it does not ensure that the final DNA-recognition preferences are the optimal ones, because of suboptimal local maxima of the likelihood function. This can be overcome by using promising starting points or applying the EM procedure with multiple random starting points (see Figure S7). An additional potential pitfall is over-fitting the recognition preferences of rare residues. To address this problem and ensure that the estimated recognition preferences for rare amino acids are close to uniform distribution (i.e., uninformative), we use a standard method of “pseudo-counts.” We do so by adding a constant (0.7 in the results above) to each amino acid–nucleotide count computed at the end of the E-step. This is equivalent to using a Dirichlet prior on the parameters, and then performing a maximum a posteriori estimation rather than maximum likelihood estimation.

We evaluated the robustness and convergence rate of the EM procedure using a 10-fold cross-validation procedure. In each round, we removed a part of the data, trained on the remaining pairs, and tested the likelihood of the held-out protein–DNA pairs. We used this procedure to test various initialization options. Our evaluation shows that the EM algorithm performs best when initialized with the general recognition preferences of Mandel-Gutfreund et al. [5], converging within a few iterations. Similar results were obtained using random initialization points, although the convergence rate was somewhat slower (see Figure S7). Also, in Figure S8 we demonstrate the correlation between the size of the training dataset and the likelihood of test data.

#### Predicting the binding sites of novel proteins.

Given the sequence of a novel Cys_2_His_2_ Zinc Finger protein, we identified the four key residues at each DNA-binding domain, and utilized the appropriate set of DNA-recognition preferences to construct a probabilistic model of the binding site (see Figure 3).

#### In silico binding experiments.

We used the predicted binding site models to scan genomic sequences for putative binding sites. We scored each possible binding position using the log of the ratio between the probability assigned to it by the model and the background probability (log-odds score). We then estimated the *p*-value of these scores and applied a Bonferroni correction to account for multiple tests within the same promoter region [38]. Sites with a significant *p*-value (≤0.05 after Bonferroni correction) were marked as putative binding sites (see Figure 4B).

#### Comparison with other computational approaches.

In a similar manner, we generated probabilistic binding site models for these transcription factors using the recognition preferences of Mandel-Gutfreund et al. [5] and SAMIE [15]. We then scanned the corresponding promoter regions using these models.

#### Ab initio genome-wide prediction of binding sites.

We downloaded genomic sequences of the *D. melanogaster* from FlyBase [22], release 3–1. These include 2-kb regulatory regions upstream from 15,664 genes, and the sequences of 16,201 putative gene products. We scanned the proteins for canonical Zinc Finger domains using the Cys_2_His_2_ conserved pattern and our profile-HMM model (available at http://compbio.cs.huji.ac.il/Zinc). We found 29 proteins with properly spaced three or four fingers (with distances of 28–31 residues between the beginnings of Zinc Finger domains). We then used the learned sets of DNA-recognition preferences to predict probabilistic binding site models for these putative Zinc Finger transcription factors. Finally, we performed in silico binding experiments by scanning each gene's 2-kb upstream region for two significant binding sites (*p* ≤ 0.05 after Bonferroni correction). The matched genes were marked as putative direct targets of the transcription factor.

#### Enrichment of GO annotations among the target genes.

FlyBase GO annotations [18,22] were downloaded from the Gene Ontology Consortium (http://www.geneontology.org) in October 2003. The enrichment *p*-values were calculated by GeneXPress (http://genexpress.stanford.edu), using a hyper-geometric test that compares the abundance of similarly annotated genes among the putative targets to the rest of the genome. We then applied an FDR correction for multiple hypotheses using a false rate of 0.05 [39], and only significant factors/terms are shown.

#### Inference of activity/function using gene expression data.

We downloaded genome-wide gene expression data from early embryogenesis stages [20] (available from FlyBase; http://www.fruitfly.org/cgi-bin/ex/insitu.pl). The expression level of each gene in each array was transformed to log (base 2) of the ratio of expression to the geometric average of the expression of the gene in all arrays. In addition, we downloaded expression data from along the *Drosophila* life cycle [21] (available from Stanford Microarray Database; http://genome-www5.stanford.edu). These expression data are represented as log (base 2) of expression compared to a reference sample representing all stages of the life cycle.

For each protein and in each experiment, we used a Kolmogorov-Smirnov test to evaluate whether the expression pattern of the putative direct target genes was different from the expression of the rest of the genome. We then corrected the results for multiple hypotheses using an FDR correction [39] (false rate of 0.05). Similarly, we used differential gene expression data from *D. melanogaster* imaginal wing disc [23]. For each gene, we computed the ratio of its expression in the body wall to its expression in the hinge-wing pouch, and performed a two-tailed version of the Kolmogorov-Smirnov test to compare these ratios among the putative targets and the rest of the genome.

## Supporting Information

Figure S1 Sequence logos of 29 *Drosophila* Transcription Factors (617 KB PDF).

Figure S2 Number of Predicted Direct Targets (162 KB PDF).

Figure S3 Percentage of Pairwise Coverage between Targets (109 KB PDF).

Figure S4 Results of Complete GO Table (182 KB PDF).

Figure S5 Abundance of DNA-Binding Residues in Training Data (123 KB PDF).

Figure S6 Abundance of Combinations of DNA-Binding Residues in Training Data (123 KB PDF).

Figure S7 Convergence of the EM Algorithm on Held-Out Test Data (106 KB PDF).

Figure S8 Likelihood of Held-Out Test Data Given Different Sizes of the Training Datasets (106 KB PDF).

Table S1 Four Sets of DNA-Recognition Preferences: Probabilities (22 KB PDF).

Table S2 Four Sets of Recognition Preferences: Counts (20 KB PDF).

Table S3 Confidence Intervals on Four Sets of DNA-Recognition Preferences (63 KB PDF).

Table S4 Correlation with Experimentally Measured Binding Affinities (514 KB TIF).

Table S5 21 Protein–DNA Pairs (2 MB TIF).

Table S6 Sensitivity and Specificity of Test Set at Different Significance Threshold Values (328 KB TIF).

Table S7 Sensitivity and Specificity of Test Set at Different Significance Threshold Values—Other Computational Methods (440 KB TIF).

Table S8 Position-Specific Score Matrices of 29 Cys_2_His_2_ Transcription Factors from *Drosophila melanogaster*
(55 KB PDF).

## Abbreviations

EM: expectation maximization
GO: Gene Ontology
