# Supplementary material for: Ab Initio Prediction of Transcription Factor Targets Using Structural Knowledge
Source: PLoS Comput Biol. 2005 Jun 24;1(1):e1. doi: 10.1371/journal.pcbi.0010001 (PMC1183507; doi:10.1371/journal.pcbi.0010001)
Supplement: Figure S1 — (617 KB PDF). [file pcbi.0010001.sg001.pdf]

Figure S1 - Seq. logos of 29 Dros. Transcription factors
The predicted binding site models of 29 Drosophila Cys_2His_2 ZF transcription factors are shown as sequences logos.

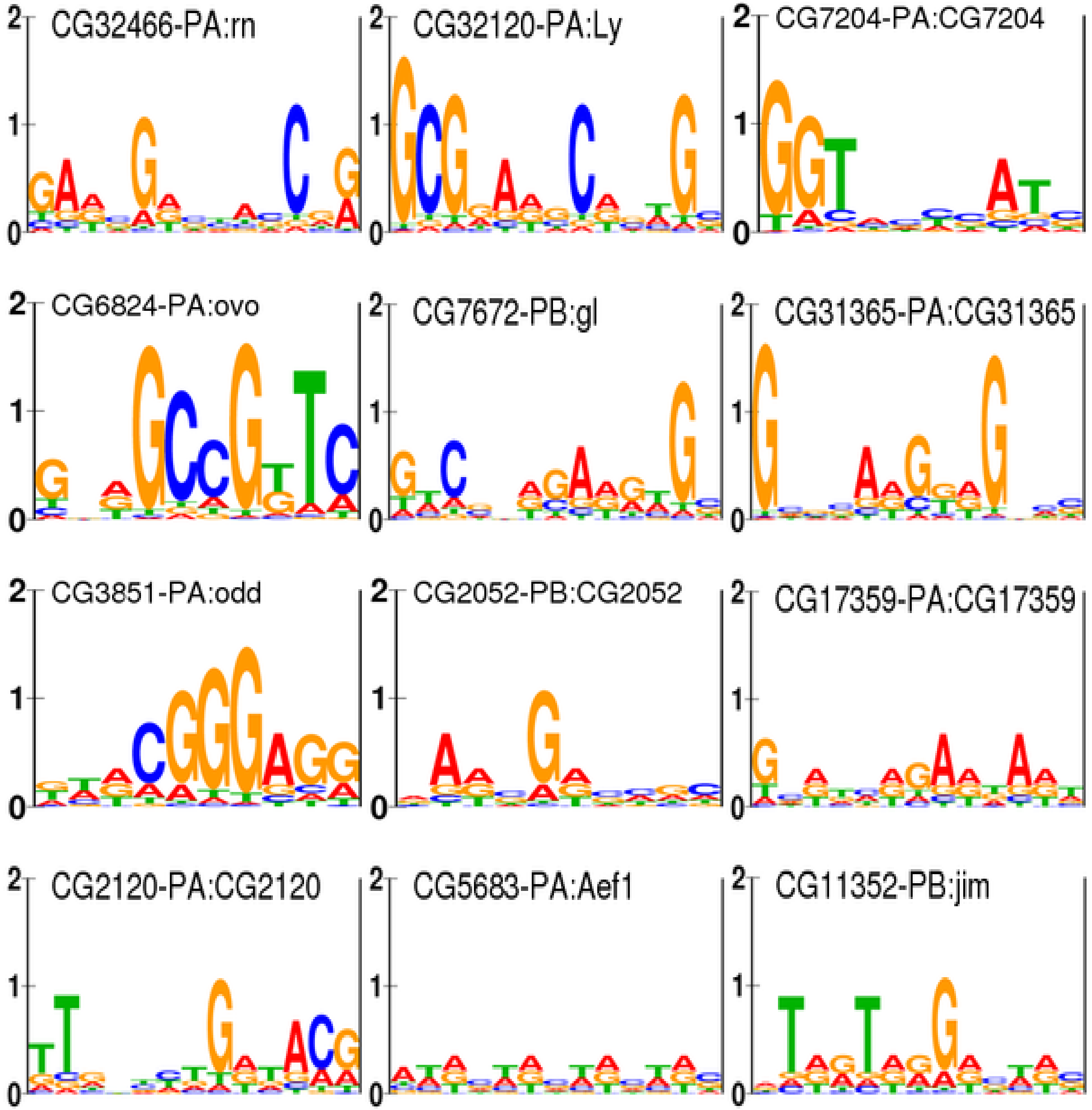

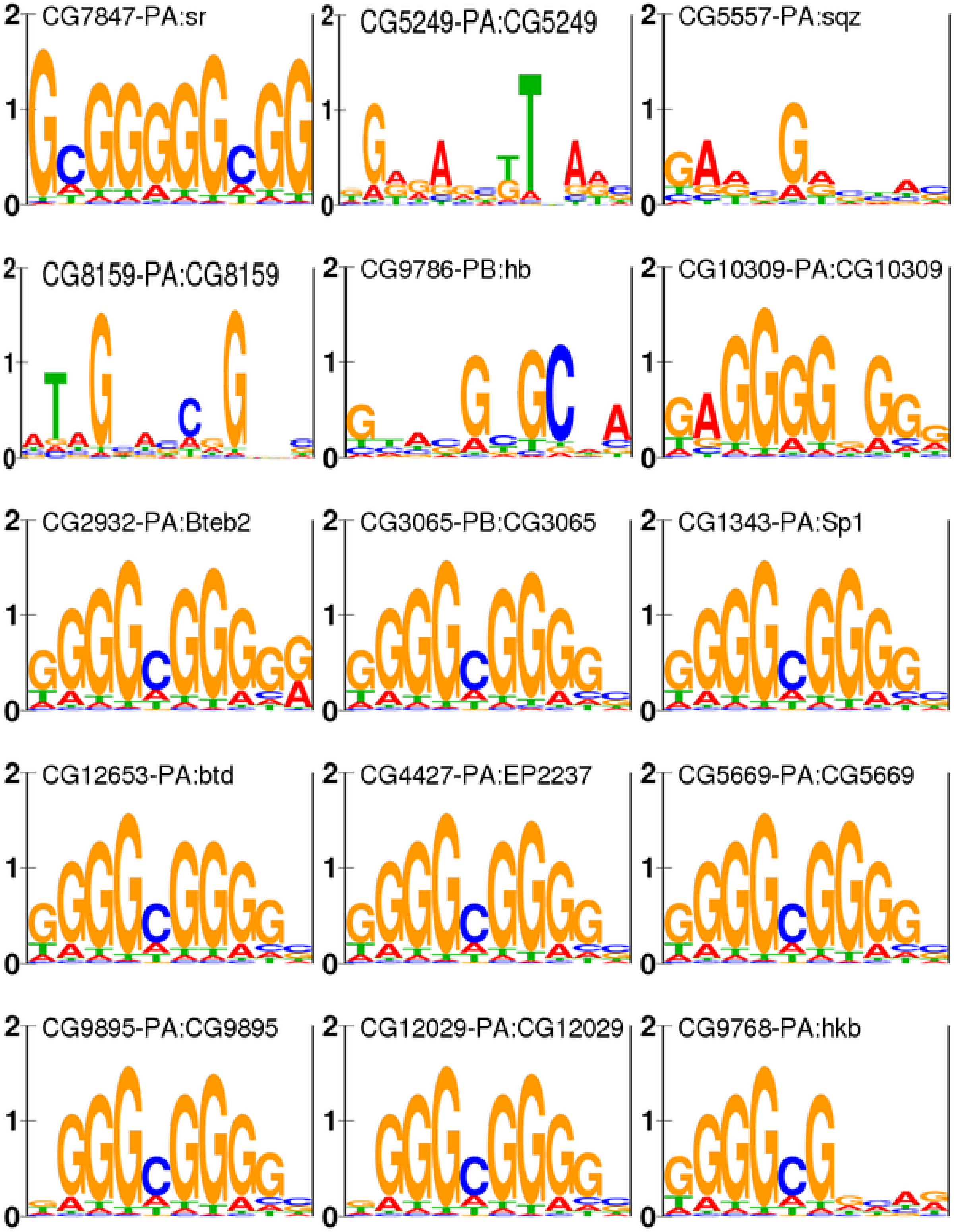
CG7847-PA:sr
CG5249-PA:CG5249
CG5557-PA:sqz
CG8159-PA:CG8159
CG9786-PB:hb
CG10309-PA:CG10309
CG2932-PA:Bteb2
CG3065-PB:CG3065
CG1343-PA:Sp1
CG12653-PA:btd
CG4427-PA:EP2237
CG5669-PA:CG5669
CG9895-PA:CG9895
CG12029-PA:CG12029
CG9768-PA:hkb

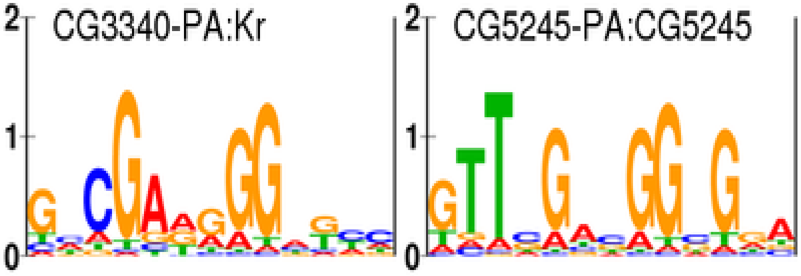
CG3340-PA:Kr
CG5245-PA:CG5245
2
1
0
