# Supplementary material for: Ab Initio Prediction of Transcription Factor Targets Using Structural Knowledge
Source: PLoS Comput Biol. 2005 Jun 24;1(1):e1. doi: 10.1371/journal.pcbi.0010001 (PMC1183507; doi:10.1371/journal.pcbi.0010001)

Figure S2 – Number of predicted direct targets

The number of predicted targets genes is shown for each transcription factor. A gene is considered to be a direct target of the factor if its 2Kb promoter region contain at least two significant putative binding sites (Bonferroni corrected p-value ≤ 0.05)

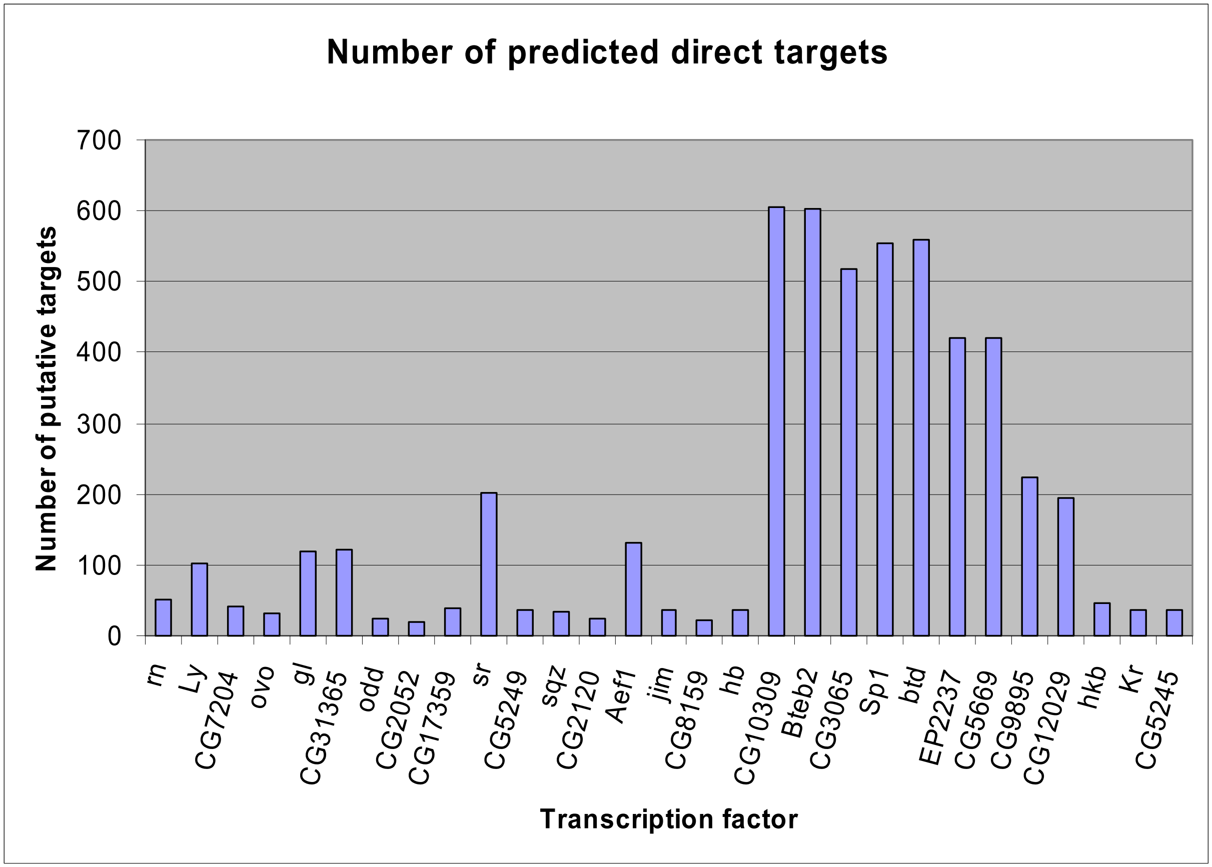

Supplement: Figure S2 — (162 KB PDF). [file pcbi.0010001.sg002.pdf]
