# Supplementary material for: Ab Initio Prediction of Transcription Factor Targets Using Structural Knowledge
Source: PLoS Comput Biol. 2005 Jun 24;1(1):e1. doi: 10.1371/journal.pcbi.0010001 (PMC1183507; doi:10.1371/journal.pcbi.0010001)

Figure S3 – Percentage of pairwise coverage between targets

For each pair of transcription factors we consider the intersection among the predicted groups of targets genes, and compute the ratios between the size of the intersection and number of targets of each TF alone.

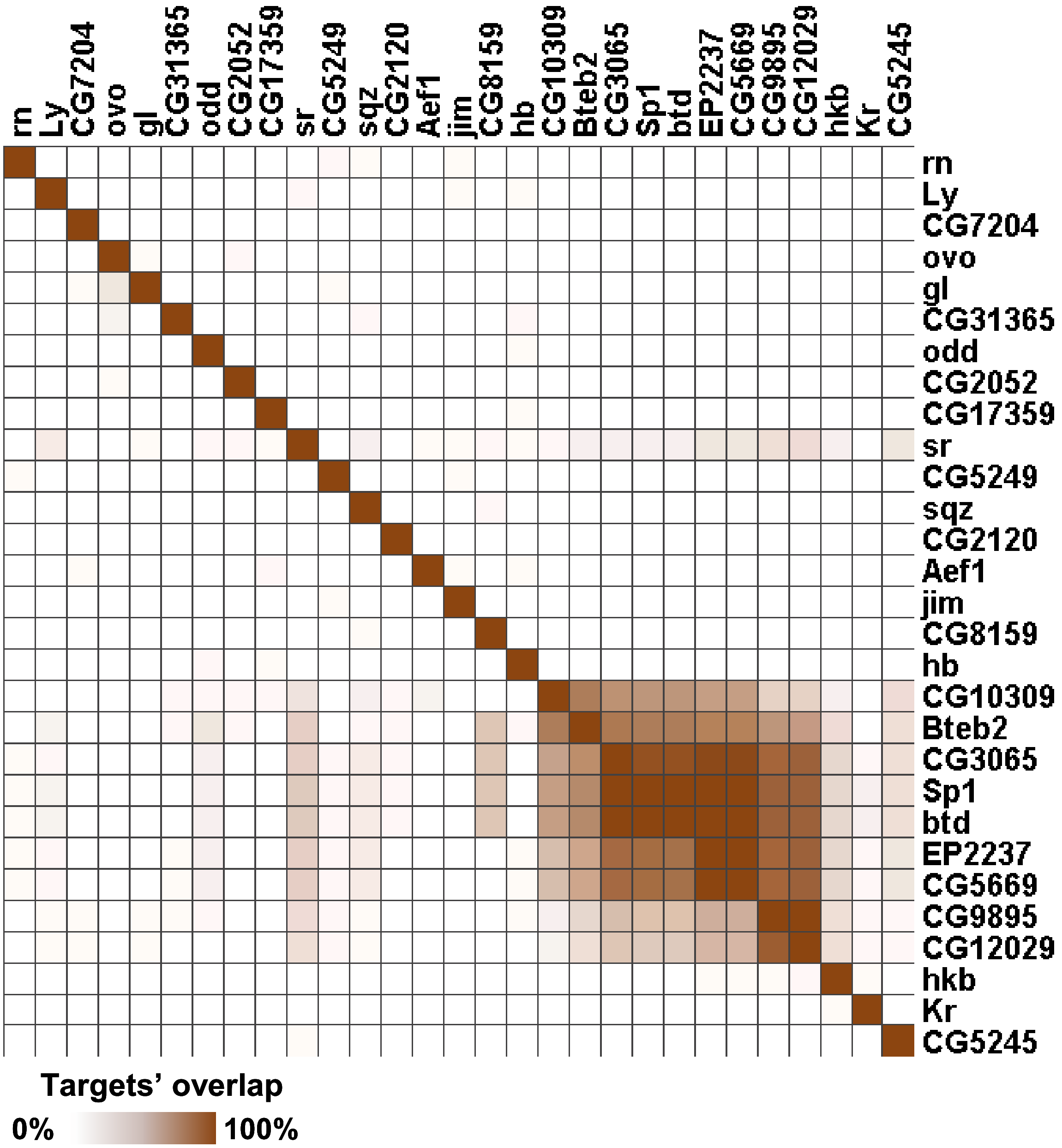

Supplement: Figure S3 — (109 KB PDF). [file pcbi.0010001.sg003.pdf]
