# Supplementary material for: Ab Initio Prediction of Transcription Factor Targets Using Structural Knowledge
Source: PLoS Comput Biol. 2005 Jun 24;1(1):e1. doi: 10.1371/journal.pcbi.0010001 (PMC1183507; doi:10.1371/journal.pcbi.0010001)

Figure S4 - Results of complete GO table

Full table of gene annotation enrichment among the putative target sets of 29 transcription factors in D. melanogaster. Blue cells correspond to significant over-abundance of a GO term (row) among the predicted targets of a protein (column), using a hyper-geometric test. The data are corrected for multiple tests using FDR and only significant cells ($p < 0.05$) are shown.

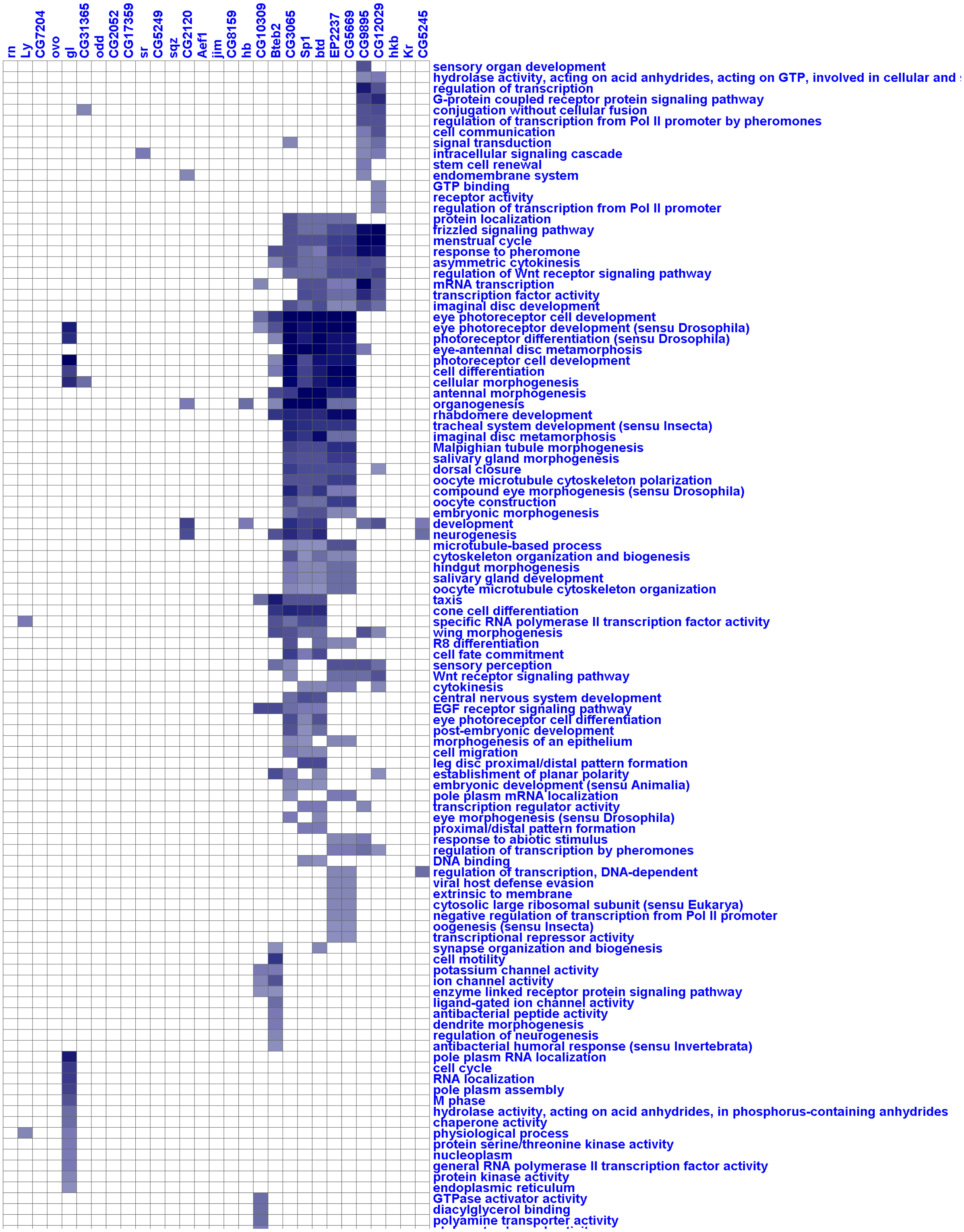

Supplement: Figure S4 — (182 KB PDF). [file pcbi.0010001.sg004.pdf]
