# Supplementary material for: Ab Initio Prediction of Transcription Factor Targets Using Structural Knowledge
Source: PLoS Comput Biol. 2005 Jun 24;1(1):e1. doi: 10.1371/journal.pcbi.0010001 (PMC1183507; doi:10.1371/journal.pcbi.0010001)

Figure S5 - Abundance of DNA-binding residues in the training data.
The training data consists of 1320 zinc fingers that interact with 455 binding sites. The abundance of each residue at each DNA-binding position is plotted in the following four figures.

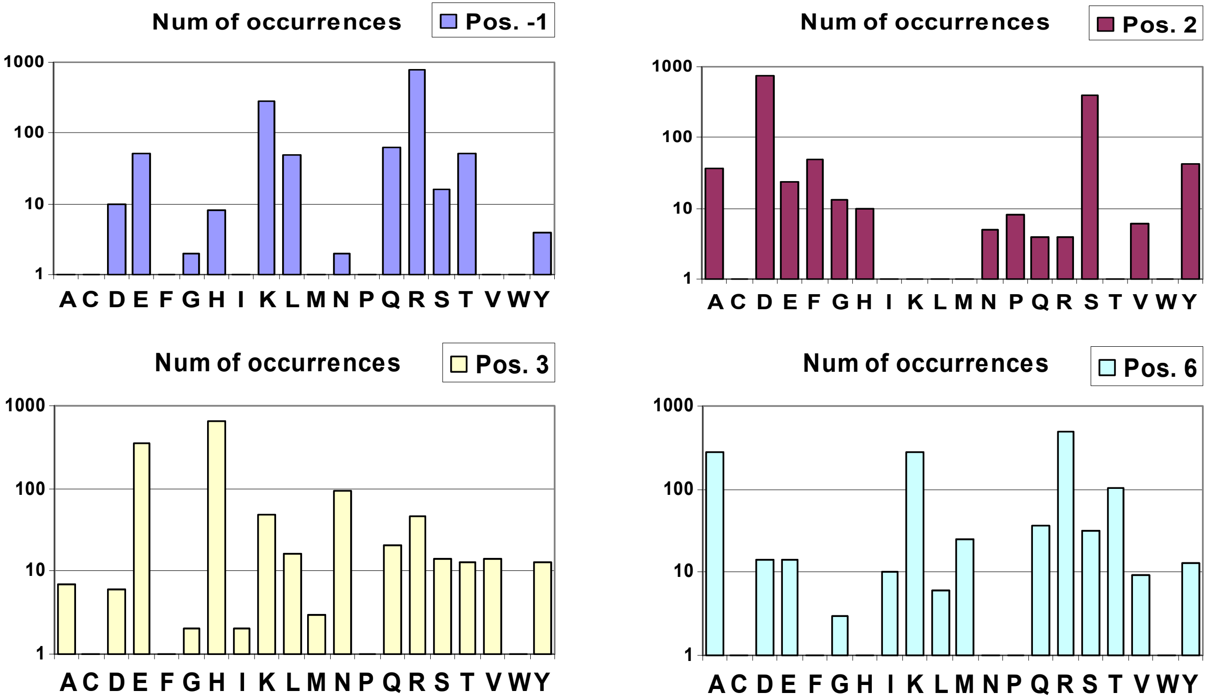

Supplement: Figure S5 — (123 KB PDF). [file pcbi.0010001.sg005.pdf]
