# Supplementary material for: Ab Initio Prediction of Transcription Factor Targets Using Structural Knowledge
Source: PLoS Comput Biol. 2005 Jun 24;1(1):e1. doi: 10.1371/journal.pcbi.0010001 (PMC1183507; doi:10.1371/journal.pcbi.0010001)

Figure S6 - Abundance of combinations of DNA-binding residues in the training data.
When considering only the 4 DNA-binding residues, there are 80 different combinations on zinc fingers in our training data. The figure on the left shows the number of occurrences for each combination (shown are 55 fingers with 2 or more occurrences). The figure on the right zooms onto the combinations with >10 occurrences.

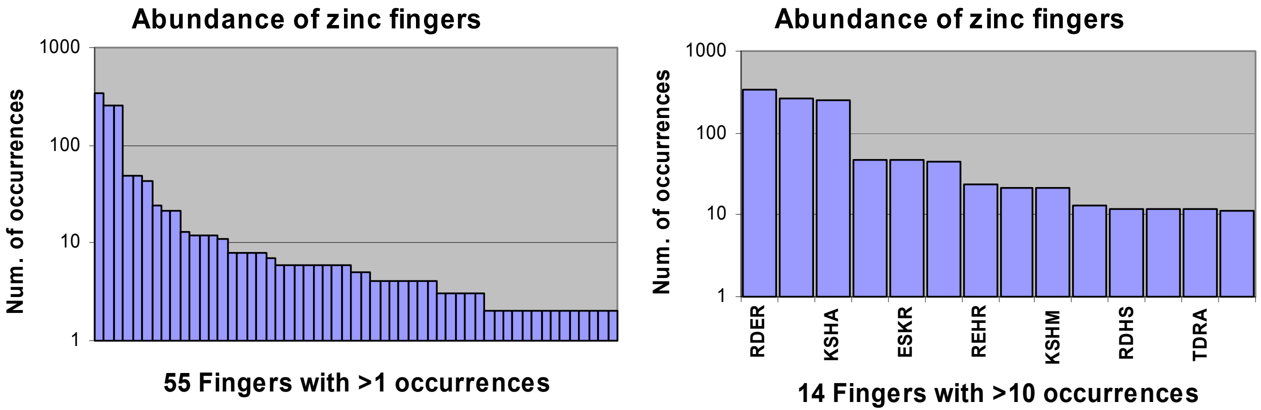

Supplement: Figure S6 — (123 KB PDF). [file pcbi.0010001.sg006.pdf]
