# Supplementary material for: Ab Initio Prediction of Transcription Factor Targets Using Structural Knowledge
Source: PLoS Comput Biol. 2005 Jun 24;1(1):e1. doi: 10.1371/journal.pcbi.0010001 (PMC1183507; doi:10.1371/journal.pcbi.0010001)

Figure S7 - Convergence of the EM algorithm on held-out test data.
10-fold cross-validation tests show a significant improvement in the average log-likelihood per interaction (in bits), along 14 EM iterations. We show the improvement of the recognition preferences for different starting points. These include the prior knowledge parameters from Mandel-Gutfreund et al. (2001) and random starting points.

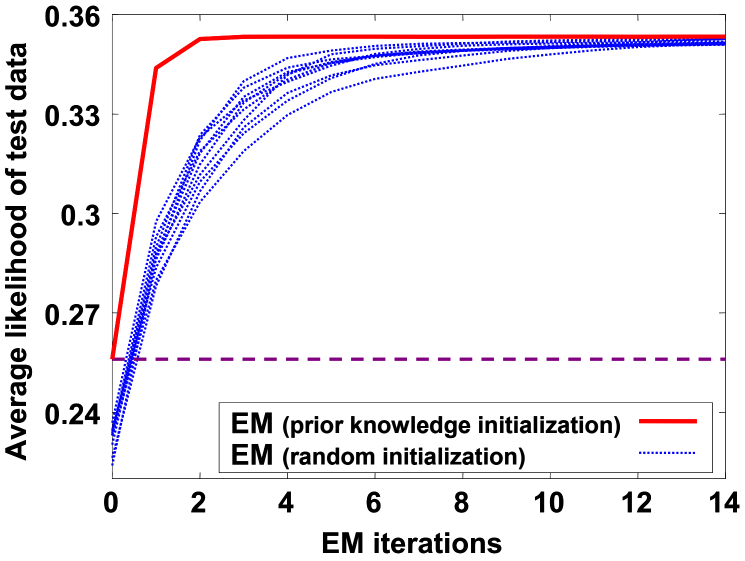

Supplement: Figure S7 — (106 KB PDF). [file pcbi.0010001.sg007.pdf]
