# Supplementary material for: Ab Initio Prediction of Transcription Factor Targets Using Structural Knowledge
Source: PLoS Comput Biol. 2005 Jun 24;1(1):e1. doi: 10.1371/journal.pcbi.0010001 (PMC1183507; doi:10.1371/journal.pcbi.0010001)

## Figure S8 - Likelihood of held-out test data given different sizes of training dataset.

The original training data (455 canonical $Cys_2His_2$ zinc finger sites from TRANSFAC 7.3) were split into 10 equally-sized sets. We used each one as held-out test data, while applying the following procedure 10 times: Various portions at different sizes (from 10 to 400 binding sites) were sampled from the remaining 90% of the data. These sites were used as training data for the EM algorithm (15 iterations). We then used the held-out data as test data, and calculated its likelihood. We then averaged the likelihood over all 10 repeats.

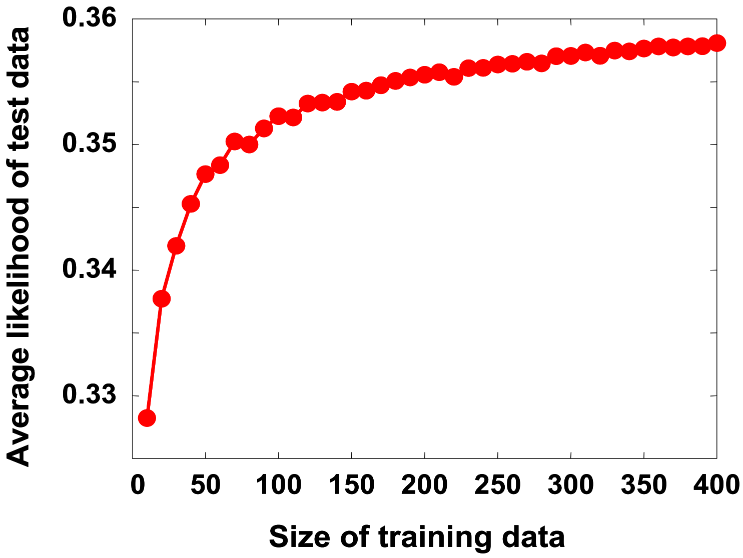

Supplement: Figure S8 — (106 KB PDF). [file pcbi.0010001.sg008.pdf]
