# Supplementary material for: Ab Initio Prediction of Transcription Factor Targets Using Structural Knowledge
Source: PLoS Comput Biol. 2005 Jun 24;1(1):e1. doi: 10.1371/journal.pcbi.0010001 (PMC1183507; doi:10.1371/journal.pcbi.0010001)
Supplement: Table S1 — (22 KB PDF). [file pcbi.0010001.st001.pdf]

Table S1 - Four sets of DNA-recognition preferences: Probabilities

Set of recognition preferences for amino acids at position 6 of the a-helix

	Gly	Ala	Val	Ile	Leu	Phe	Trp	Tyr	Met	Cys	Thr	Ser	Gln	Asn	Glu	Asp	His	Arg	Lys	Pro
A	0.297	0.01	0.232	0.124	0.192	0.121	0.25	0.039	0.145	0.293	0.224	0.074	0.32	0.524	0.146	0.103	0.23	0.011	0.111	0.146
C	0.125	0.003	0.053	0.056	0.102	0.466	0.25	0.136	0.104	0.431	0.097	0.116	0.112	0.169	0.566	0.674	0.108	0.012	0.035	0.146
G	0.125	0.884	0.364	0.227	0.41	0.121	0.25	0.632	0.337	0.155	0.53	0.661	0.316	0.139	0.23	0.108	0.427	0.948	0.664	0.146
T	0.453	0.104	0.352	0.593	0.296	0.293	0.25	0.193	0.413	0.121	0.149	0.149	0.252	0.169	0.057	0.115	0.235	0.029	0.19	0.563

Set of recognition preferences for amino acids at position 3 of the a-helix

	Gly	Ala	Val	Ile	Leu	Phe	Trp	Tyr	Met	Cys	Thr	Ser	Gln	Asn	Glu	Asp	His	Arg	Lys	Pro
A	0.189	0.048	0.074	0.073	0.059	0.121	0.25	0.124	0.204	0.354	0.35	0.242	0.317	0.705	0.173	0.046	0.142	0.15	0.062	0.146
C	0.432	0.085	0.103	0.072	0.049	0.466	0.25	0.345	0.285	0.354	0.16	0.201	0.379	0.085	0.66	0.858	0.027	0.01	0.016	0.146
G	0.195	0.083	0.44	0.073	0.386	0.121	0.25	0.174	0.084	0.146	0.064	0.328	0.083	0.139	0.025	0.047	0.804	0.704	0.816	0.146
T	0.185	0.784	0.384	0.783	0.506	0.293	0.25	0.357	0.426	0.146	0.426	0.228	0.221	0.071	0.142	0.049	0.027	0.136	0.106	0.563

Set of recognition preferences for amino acids at position 2 of the a-helix

	Gly	Ala	Val	Ile	Leu	Phe	Trp	Tyr	Met	Cys	Thr	Ser	Gln	Asn	Glu	Asp	His	Arg	Lys	Pro
A	0.158	0.028	0.079	0.08	0.146	0.015	0.25	0.111	0.25	0.354	0.367	0.132	0.477	0.495	0.129	0.049	0.185	0.076	0.095	0.092
C	0.417	0.608	0.112	0.08	0.146	0.933	0.25	0.63	0.25	0.354	0.158	0.326	0.155	0.187	0.543	0.928	0.122	0.012	0.02	0.155
G	0.068	0.018	0.144	0.08	0.146	0.015	0.25	0.026	0.103	0.146	0.065	0.422	0.155	0.159	0.088	0.011	0.489	0.735	0.762	0.367
T	0.357	0.346	0.665	0.761	0.563	0.037	0.25	0.233	0.397	0.146	0.41	0.12	0.213	0.16	0.24	0.012	0.204	0.177	0.123	0.385

Set of recognition preferences for amino acids at position -1 relatively to the a-helix

	Gly	Ala	Val	Ile	Leu	Phe	Trp	Tyr	Met	Cys	Thr	Ser	Gln	Asn	Glu	Asp	His	Arg	Lys	Pro
A	0.247	0.055	0.121	0.08	0.072	0.121	0.428	0.202	0.25	0.354	0.38	0.233	0.402	0.529	0.218	0.129	0.165	0.044	0.095	0.146
C	0.248	0.127	0.121	0.08	0.019	0.466	0.185	0.399	0.25	0.354	0.15	0.14	0.015	0.165	0.685	0.726	0.223	0.023	0.101	0.146
G	0.258	0.056	0.121	0.08	0.017	0.121	0.198	0.071	0.103	0.146	0.213	0.48	0.338	0.138	0.031	0.068	0.408	0.878	0.71	0.146
T	0.247	0.762	0.638	0.761	0.892	0.293	0.188	0.327	0.397	0.146	0.257	0.147	0.246	0.168	0.066	0.077	0.204	0.055	0.093	0.563
