# Supplementary material for: Ab Initio Prediction of Transcription Factor Targets Using Structural Knowledge
Source: PLoS Comput Biol. 2005 Jun 24;1(1):e1. doi: 10.1371/journal.pcbi.0010001 (PMC1183507; doi:10.1371/journal.pcbi.0010001)
Supplement: Table S2 — (20 KB PDF). [file pcbi.0010001.st002.pdf]

Table S2 - Four sets of recognition preferences: Counts

Count table of amino acids-nucleotide interactions at position 6 of the a-helix

	Gly	Ala	Val	Ile	Leu	Phe	Trp	Tyr	Met	Cys	Thr	Ser	Gln	Asn	Glu	Asp	His	Arg	Lys	Pro
A	1.69	2.63	3.11	1.62	1.82	0.7	0.7	0.77	2.71	1.7	22.66	3.18	9.58	17.7	3.22	1.64	3.62	5.71	28.46	0.7
C	0.71	0.81	0.71	0.73	0.97	2.7	0.7	2.71	1.94	2.5	9.86	5.03	3.36	5.7	12.46	10.77	1.7	6.11	9.09	0.7
G	0.71	237.29	4.89	2.98	3.89	0.7	0.7	12.61	6.3	0.9	53.75	28.56	9.45	4.7	5.07	1.73	6.73	491.15	170.77	0.7
T	2.57	27.81	4.72	7.78	2.81	1.7	0.7	3.85	7.72	0.7	15.07	6.46	7.54	5.7	1.26	1.84	3.7	14.88	48.97	2.7

Count table of amino acids-nucleotide interactions at position 3 of the a-helix

	Gly	Ala	Val	Ile	Leu	Phe	Trp	Tyr	Met	Cys	Thr	Ser	Gln	Asn	Glu	Asp	His	Arg	Lys	Pro
A	0.73	0.71	1.21	0.71	0.85	0.7	0.7	1.65	1.73	1.7	6.45	5.8	11.1	74.74	58.78	0.73	85.56	14.41	4.84	0.7
C	1.66	1.26	1.67	0.7	0.71	2.7	0.7	4.58	2.41	1.7	2.96	4.81	13.26	9.04	223.91	13.57	15.94	0.98	1.27	0.7
G	0.75	1.24	7.17	0.72	5.57	0.7	0.7	2.31	0.71	0.7	1.18	7.86	2.91	14.77	8.65	0.74	483.21	67.81	63.57	0.7
T	0.71	11.62	6.26	7.66	7.3	1.7	0.7	4.74	3.61	0.7	7.85	5.47	7.75	7.5	48.08	0.77	16.06	13.12	8.26	2.7

Count table of amino acids-nucleotide interactions at position 2 of the a-helix

	Gly	Ala	Val	Ile	Leu	Phe	Trp	Tyr	Met	Cys	Thr	Ser	Gln	Asn	Glu	Asp	His	Arg	Lys	Pro
A	2.21	1.14	0.74	0.7	0.7	0.71	0.7	3.81	1.7	1.7	3.96	36.41	8.51	17.74	3.28	33.64	3.72	4.71	3.7	0.76
C	5.81	24.89	1.05	0.7	0.7	43.28	0.7	21.57	1.7	1.7	1.7	89.81	2.77	6.71	13.82	642.83	2.45	0.76	0.77	1.28
G	0.94	0.75	1.35	0.7	0.7	0.7	0.7	0.9	0.7	0.7	0.7	116.25	2.76	5.69	2.24	7.69	9.8	45.71	29.63	3.02
T	4.97	14.16	6.24	6.7	2.7	1.71	0.7	7.98	2.7	0.7	4.43	33.13	3.79	5.72	6.1	8.48	4.1	10.97	4.77	3.17

Count table of amino acids-nucleotide interactions at position -1 relatively to the a-helix

	Gly	Ala	Val	Ile	Leu	Phe	Trp	Tyr	Met	Cys	Thr	Ser	Gln	Asn	Glu	Asp	His	Arg	Lys	Pro
A	0.7	0.7	0.7	0.7	3.18	0.7	1.63	2.34	1.7	1.7	16.73	6.25	22.79	18.52	9.5	2.25	3.46	35.33	24.64	0.7
C	0.7	1.6	0.7	0.7	0.82	2.7	0.7	4.63	1.7	1.7	6.6	3.78	0.83	5.79	29.9	12.67	4.67	18.03	26.3	0.7
G	0.73	0.71	0.7	0.7	0.77	0.7	0.75	0.82	0.7	0.7	9.35	12.91	19.16	4.82	1.34	1.19	8.56	699.73	184.07	0.7
T	0.7	9.66	3.7	6.7	39.52	1.7	0.71	3.79	2.7	0.7	11.3	3.95	13.92	5.86	2.89	1.34	4.29	44.03	24.14	2.7
