# Supplementary material for: Ab Initio Prediction of Transcription Factor Targets Using Structural Knowledge
Source: PLoS Comput Biol. 2005 Jun 24;1(1):e1. doi: 10.1371/journal.pcbi.0010001 (PMC1183507; doi:10.1371/journal.pcbi.0010001)
Supplement: Table S3 — (63 KB PDF). [file pcbi.0010001.st003.pdf]

Table S3 – Confidence intervals on four sets of DNA-recognition preferences:

10-90% confidence intervals of recognition preferences for amino acids at position 6 of the a-helix

	Gly	Ala	Val	Ile	Leu	Phe	Trp	Tyr	Met	Cys	Thr	Ser	Gln	Asn	Glu	Asp	His	Arg	Lys	Pro
A	0.084 - 0.548	0.003 - 0.018	0.098 - 0.384	0.029 - 0.245	0.053 - 0.362	0.007 - 0.298	0.017 - 0.590	0.003 - 0.095	0.054 - 0.254	0.083 - 0.542	0.172 - 0.277	0.029 - 0.127	0.214 - 0.431	0.414 - 0.633	0.061 - 0.247	0.024 - 0.204	0.105 - 0.370	0.006 - 0.017	0.086 - 0.136	0.008 - 0.358
C	0.007 - 0.307	0.000 - 0.007	0.003 - 0.131	0.003 - 0.138	0.011 - 0.232	0.212 - 0.725	0.017 - 0.590	0.050 - 0.238	0.029 - 0.199	0.183 - 0.692	0.062 - 0.136	0.059 - 0.181	0.047 - 0.189	0.092 - 0.254	0.430 - 0.699	0.521 - 0.817	0.026 - 0.213	0.006 - 0.018	0.022 - 0.051	0.008 - 0.358
G	0.007 - 0.308	0.858 - 0.908	0.204 - 0.535	0.094 - 0.380	0.215 - 0.615	0.007 - 0.298	0.017 - 0.590	0.492 - 0.766	0.203 - 0.480	0.016 - 0.354	0.467 - 0.594	0.567 - 0.751	0.211 - 0.426	0.069 - 0.218	0.123 - 0.349	0.027 - 0.212	0.272 - 0.587	0.936 - 0.960	0.626 - 0.701	0.008 - 0.358
T	0.199 - 0.716	0.081 - 0.128	0.193 - 0.521	0.419 - 0.761	0.123 - 0.490	0.083 - 0.541	0.017 - 0.590	0.090 - 0.310	0.271 - 0.560	0.007 - 0.298	0.105 - 0.195	0.085 - 0.221	0.155 - 0.356	0.092 - 0.254	0.009 - 0.123	0.030 - 0.222	0.110 - 0.376	0.020 - 0.038	0.160 - 0.222	0.278 - 0.833

10-90% confidence intervals of recognition preferences for amino acids at position 3 of the a-helix

	Gly	Ala	Val	Ile	Leu	Phe	Trp	Tyr	Met	Cys	Thr	Ser	Gln	Asn	Glu	Asp	His	Arg	Lys	Pro
A	0.013 - 0.452	0.003 - 0.119	0.011 - 0.160	0.004 - 0.180	0.005 - 0.141	0.007 - 0.298	0.017 - 0.590	0.030 - 0.245	0.055 - 0.389	0.106 - 0.636	0.213 - 0.494	0.137 - 0.358	0.219 - 0.419	0.647 - 0.760	0.147 - 0.200	0.003 - 0.114	0.125 - 0.161	0.105 - 0.197	0.031 - 0.099	0.008 - 0.358
C	0.137 - 0.747	0.014 - 0.181	0.025 - 0.203	0.004 - 0.178	0.003 - 0.122	0.212 - 0.725	0.017 - 0.590	0.186 - 0.515	0.106 - 0.489	0.106 - 0.636	0.063 - 0.274	0.104 - 0.309	0.276 - 0.484	0.053 - 0.121	0.627 - 0.692	0.741 - 0.955	0.019 - 0.035	0.001 - 0.024	0.003 - 0.035	0.008 - 0.358
G	0.014 - 0.462	0.013 - 0.179	0.286 - 0.597	0.004 - 0.181	0.228 - 0.552	0.007 - 0.298	0.017 - 0.590	0.058 - 0.312	0.005 - 0.209	0.008 - 0.358	0.009 - 0.139	0.210 - 0.453	0.031 - 0.146	0.098 - 0.183	0.015 - 0.037	0.003 - 0.115	0.783 - 0.825	0.644 - 0.762	0.758 - 0.870	0.008 - 0.358
T	0.012 - 0.447	0.642 - 0.908	0.235 - 0.539	0.608 - 0.930	0.339 - 0.672	0.083 - 0.541	0.017 - 0.590	0.197 - 0.528	0.218 - 0.644	0.008 - 0.358	0.282 - 0.574	0.126 - 0.342	0.136 - 0.314	0.041 - 0.104	0.118 - 0.166	0.003 - 0.119	0.019 - 0.035	0.094 - 0.182	0.064 - 0.152	0.278 - 0.833

10-90% confidence intervals of recognition preferences for amino acids at position 2 of the a-helix

	Gly	Ala	Val	Ile	Leu	Phe	Trp	Tyr	Met	Cys	Thr	Ser	Gln	Asn	Glu	Asp	His	Arg	Lys	Pro
A	0.051 - 0.289	0.004 - 0.062	0.005 - 0.193	0.004 - 0.198	0.008 - 0.358	0.001 - 0.038	0.017 - 0.590	0.050 - 0.183	0.068 - 0.470	0.106 - 0.636	0.189 - 0.557	0.107 - 0.159	0.328 - 0.628	0.388 - 0.601	0.053 - 0.218	0.038 - 0.059	0.084 - 0.300	0.037 - 0.121	0.042 - 0.158	0.006 - 0.224
C	0.253 - 0.587	0.510 - 0.704	0.014 - 0.248	0.004 - 0.198	0.008 - 0.358	0.884 - 0.974	0.017 - 0.590	0.523 - 0.733	0.068 - 0.470	0.106 - 0.636	0.040 - 0.306	0.290 - 0.362	0.059 - 0.269	0.109 - 0.273	0.417 - 0.668	0.915 - 0.940	0.041 - 0.219	0.001 - 0.030	0.001 - 0.048	0.028 - 0.324
G	0.007 - 0.156	0.001 - 0.045	0.028 - 0.297	0.004 - 0.198	0.008 - 0.358	0.001 - 0.038	0.017 - 0.590	0.002 - 0.062	0.006 - 0.255	0.008 - 0.358	0.003 - 0.162	0.384 - 0.460	0.058 - 0.269	0.086 - 0.239	0.027 - 0.163	0.006 - 0.016	0.347 - 0.631	0.663 - 0.805	0.673 - 0.846	0.166 - 0.585
T	0.200 - 0.524	0.253 - 0.442	0.465 - 0.849	0.571 - 0.922	0.278 - 0.833	0.009 - 0.074	0.017 - 0.590	0.145 - 0.328	0.172 - 0.638	0.008 - 0.358	0.226 - 0.603	0.096 - 0.146	0.099 - 0.341	0.087 - 0.241	0.138 - 0.351	0.007 - 0.018	0.099 - 0.324	0.117 - 0.240	0.061 - 0.193	0.181 - 0.604

10-90% confidence intervals of recognition preferences for amino acids at position -1 relatively to the a-helix

	Gly	Ala	Val	Ile	Leu	Phe	Trp	Tyr	Met	Cys	Thr	Ser	Gln	Asn	Glu	Asp	His	Arg	Lys	Pro
A	0.017 - 0.584	0.003 - 0.138	0.007 - 0.298	0.004 - 0.198	0.028 - 0.124	0.007 - 0.298	0.133 - 0.746	0.070 - 0.359	0.068 - 0.470	0.106 - 0.636	0.288 - 0.475	0.134 - 0.340	0.319 - 0.486	0.421 - 0.636	0.141 - 0.300	0.041 - 0.236	0.072 - 0.272	0.035 - 0.054	0.073 - 0.119	0.008 - 0.358
C	0.017 - 0.585	0.029 - 0.251	0.007 - 0.298	0.004 - 0.198	0.001 - 0.045	0.212 - 0.725	0.011 - 0.449	0.222 - 0.585	0.068 - 0.470	0.106 - 0.636	0.086 - 0.221	0.063 - 0.230	0.001 - 0.035	0.091 - 0.249	0.594 - 0.773	0.586 - 0.854	0.115 - 0.343	0.016 - 0.030	0.078 - 0.126	0.008 - 0.358
G	0.019 - 0.599	0.003 - 0.140	0.007 - 0.298	0.004 - 0.198	0.001 - 0.043	0.007 - 0.298	0.015 - 0.469	0.005 - 0.170	0.006 - 0.255	0.008 - 0.358	0.137 - 0.294	0.358 - 0.603	0.259 - 0.419	0.069 - 0.215	0.005 - 0.065	0.010 - 0.148	0.274 - 0.546	0.863 - 0.892	0.674 - 0.746	0.008 - 0.358
T	0.017 - 0.584	0.604 - 0.901	0.380 - 0.870	0.571 - 0.922	0.830 - 0.946	0.083 - 0.541	0.012 - 0.454	0.162 - 0.507	0.172 - 0.638	0.008 - 0.358	0.176 - 0.343	0.068 - 0.238	0.175 - 0.320	0.092 - 0.251	0.025 - 0.117	0.014 - 0.162	0.101 - 0.321	0.045 - 0.066	0.071 - 0.117	0.278 - 0.833
