# Supplementary figures and images for: Ab Initio Prediction of Transcription Factor Targets Using Structural Knowledge
Source: PLoS Comput Biol. 2005 Jun 24;1(1):e1. doi: 10.1371/journal.pcbi.0010001 (PMC1183507; doi:10.1371/journal.pcbi.0010001)

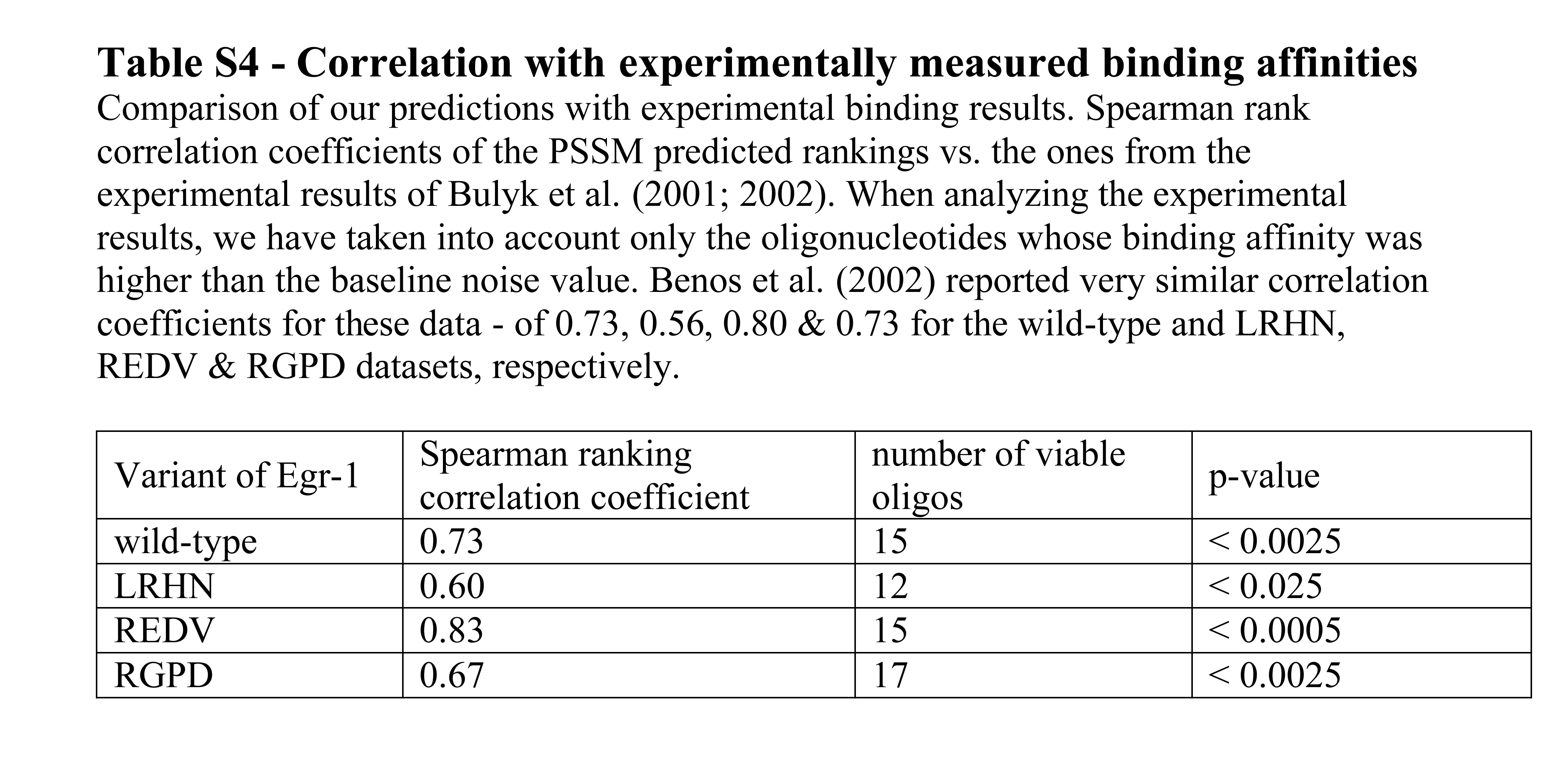

Supplement: Table S4 — (514 KB TIF). [file pcbi.0010001.st004.tif]

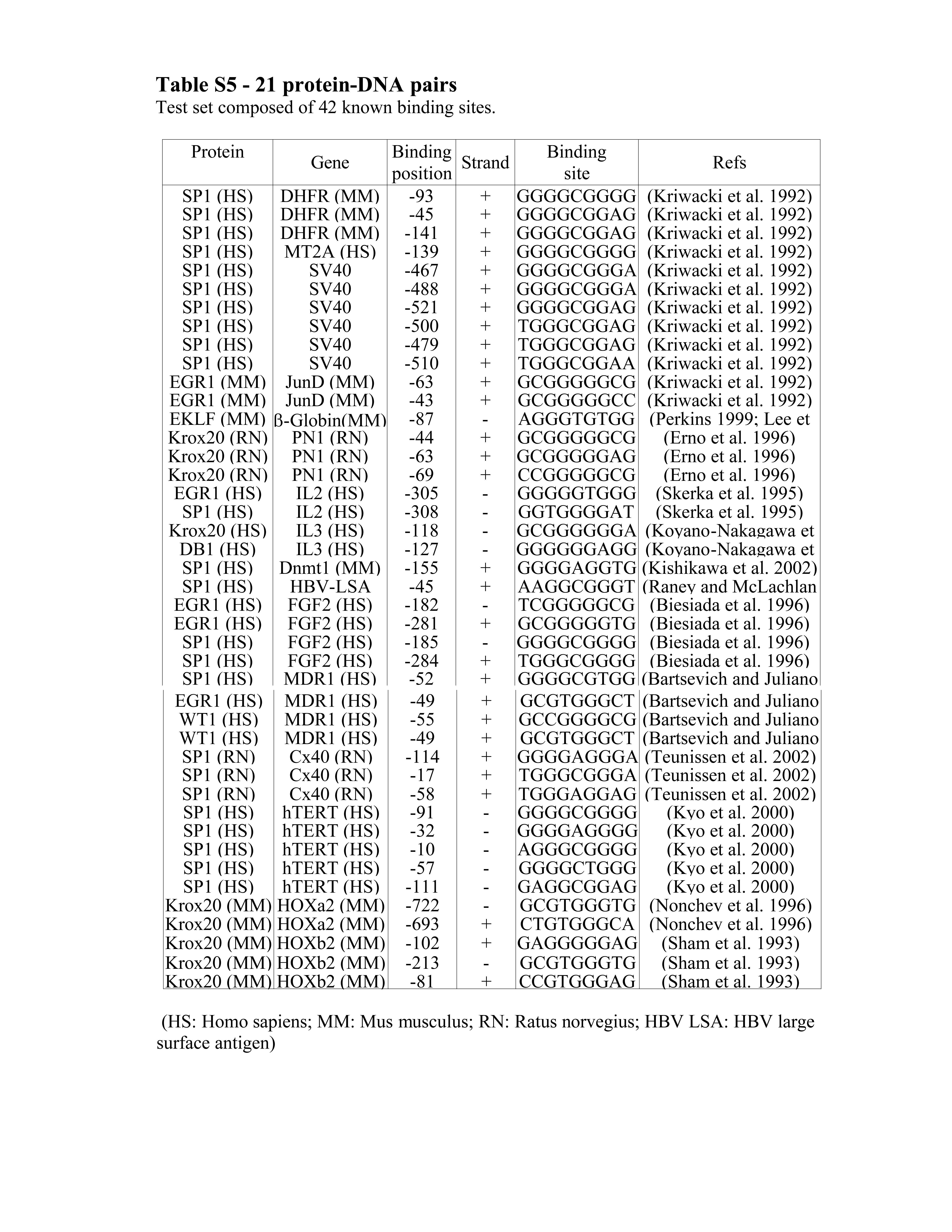

Supplement: Table S5 — (2 MB TIF). [file pcbi.0010001.st005.tif]

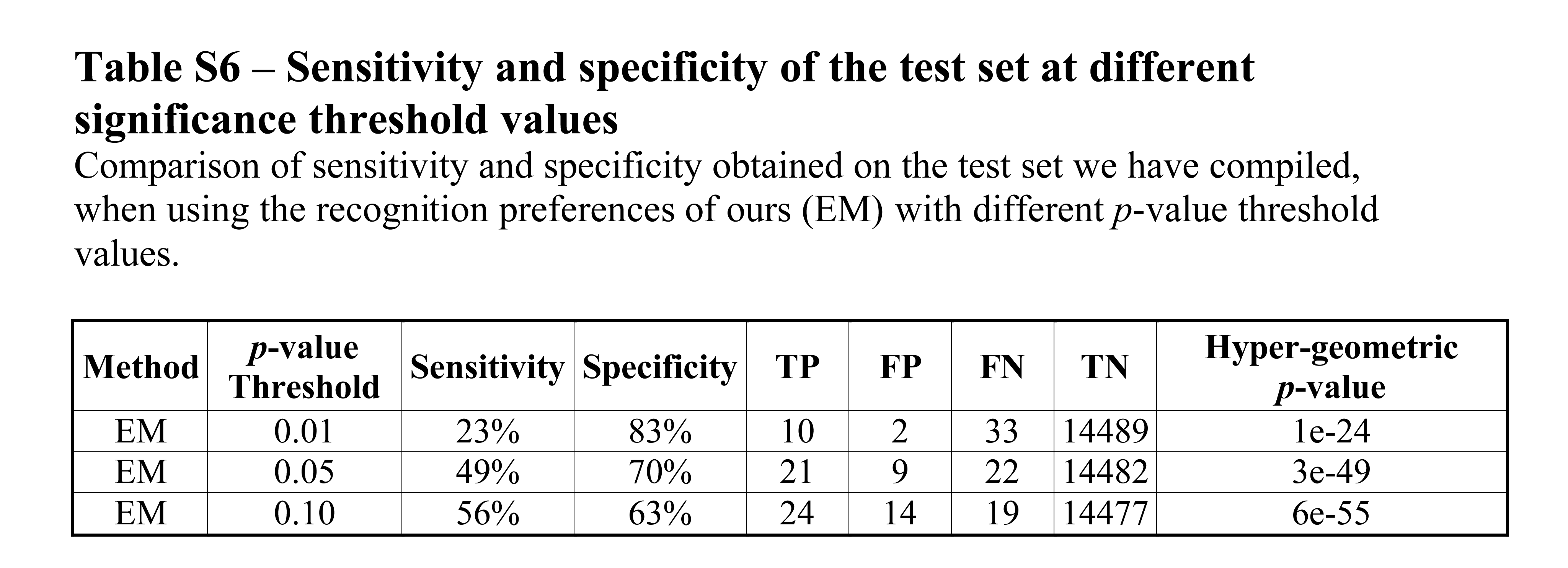

Supplement: Table S6 — (328 KB TIF). [file pcbi.0010001.st006.tif]

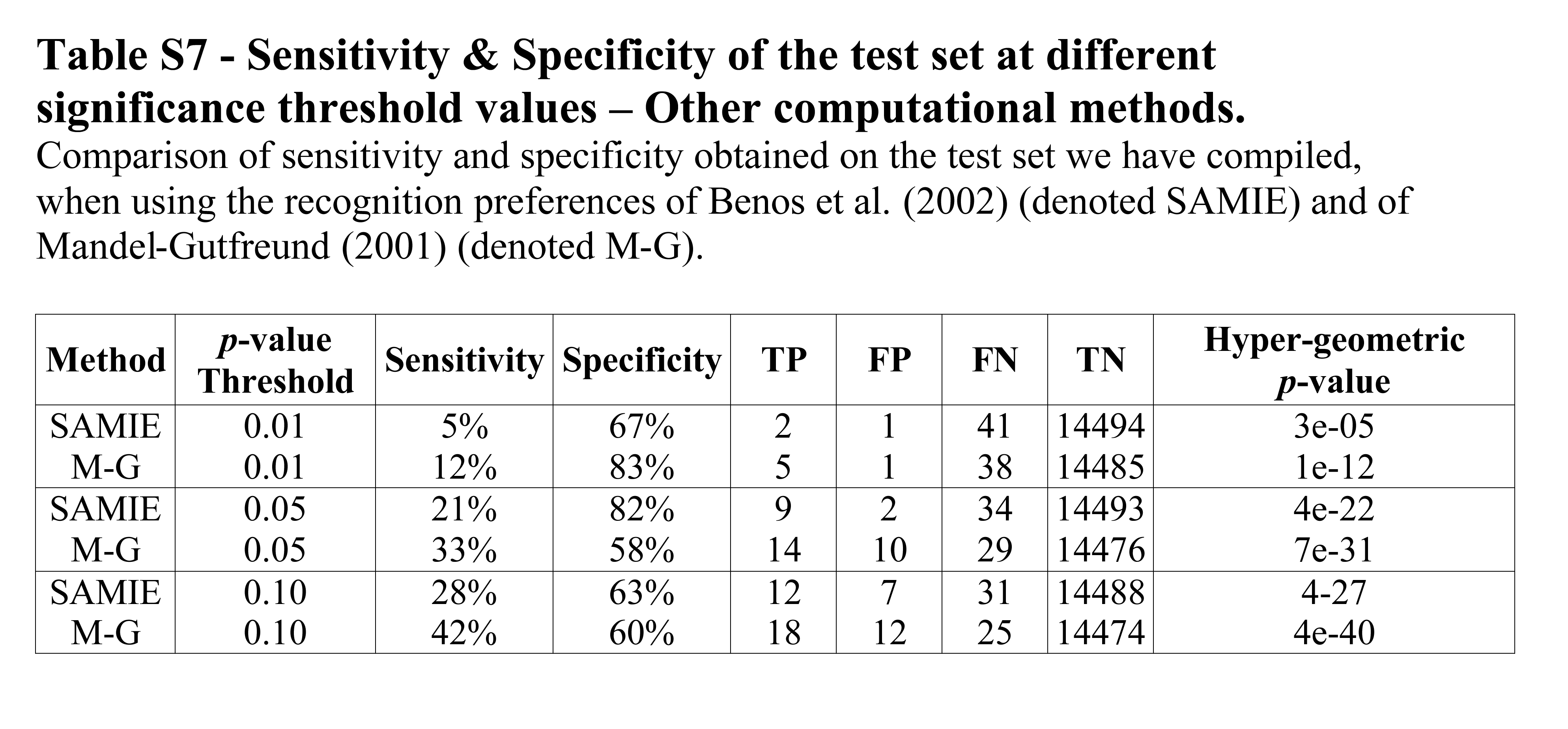

Supplement: Table S7 — (440 KB TIF). [file pcbi.0010001.st007.tif]
