# Supplementary material for: Ab Initio Prediction of Transcription Factor Targets Using Structural Knowledge
Source: PLoS Comput Biol. 2005 Jun 24;1(1):e1. doi: 10.1371/journal.pcbi.0010001 (PMC1183507; doi:10.1371/journal.pcbi.0010001)
Supplement: Table S8 — (55 KB PDF). [file pcbi.0010001.st008.pdf]

Table S8 - PSSMs of 29 Cys_2His_2 transcription factors from *Drosophila melanogaster*

CG10309-PA:CG10309

0.111	0.705	0.044	0.012	0.142	0.044	0.283	0.142	0.095	0.24
0.035	0.085	0.023	0.011	0.027	0.023	0.101	0.027	0.102	0.088
0.664	0.139	0.878	0.937	0.804	0.878	0.42	0.804	0.71	0.543
0.19	0.071	0.055	0.04	0.027	0.055	0.196	0.027	0.093	0.129

CG11352-PB:jim

0.32	0.073	0.402	0.335	0.073	0.402	0.335	0.142	0.402	0.14	0.35	0.402	0.12
0.112	0.072	0.015	0.058	0.072	0.015	0.058	0.027	0.015	0.392	0.16	0.015	0.422
0.316	0.073	0.338	0.485	0.073	0.338	0.485	0.804	0.338	0.325	0.064	0.338	0.326
0.252	0.783	0.246	0.123	0.783	0.246	0.123	0.027	0.246	0.144	0.426	0.246	0.132

CG12029-PA:CG12029

0.192	0.142	0.044	0.012	0.173	0.044	0.012	0.142	0.095	0.12
0.102	0.027	0.023	0.011	0.66	0.023	0.009	0.027	0.102	0.422
0.411	0.804	0.878	0.937	0.026	0.878	0.916	0.804	0.71	0.326
0.296	0.027	0.055	0.04	0.142	0.055	0.064	0.027	0.093	0.132

CG12653-PA:btd

0.111	0.142	0.044	0.012	0.173	0.044	0.039	0.142	0.095	0.12
0.035	0.027	0.023	0.011	0.66	0.023	0.022	0.027	0.102	0.422
0.664	0.804	0.878	0.937	0.026	0.878	0.877	0.804	0.71	0.326
0.19	0.027	0.055	0.04	0.142	0.055	0.061	0.027	0.093	0.132

CG1343-PA:Sp1

0.111	0.142	0.044	0.012	0.173	0.044	0.012	0.142	0.095	0.12
0.035	0.027	0.023	0.011	0.66	0.023	0.009	0.027	0.102	0.422
0.664	0.804	0.878	0.937	0.026	0.878	0.916	0.804	0.71	0.326
0.19	0.027	0.055	0.04	0.142	0.055	0.064	0.027	0.093	0.132

CG17359-PA:CG17359

0.111	0.189	0.402	0.179	0.317	0.402	0.143	0.705	0.402	0.244	0.705	0.402	0.213
0.035	0.432	0.015	0.13	0.379	0.015	0.104	0.085	0.015	0.062	0.085	0.015	0.155
0.664	0.195	0.338	0.235	0.083	0.338	0.601	0.139	0.338	0.325	0.139	0.338	0.155
0.19	0.185	0.246	0.456	0.221	0.246	0.152	0.071	0.246	0.369	0.071	0.246	0.477

CG2052-PB:CG2052

0.32	0.705	0.402	0.14	0.142	0.402	0.14	0.317	0.233	0.204
0.112	0.085	0.015	0.392	0.027	0.015	0.392	0.379	0.141	0.489
0.316	0.139	0.338	0.325	0.804	0.338	0.325	0.083	0.48	0.122
0.252	0.071	0.246	0.144	0.027	0.246	0.144	0.221	0.147	0.185

CG2120-PA:CG2120

0.124	0.048	0.233	0.215	0.124	0.202	0.15	0.062	0.402	0.15	0.705	0.218	0.233
0.056	0.085	0.141	0.314	0.345	0.399	0.131	0.016	0.015	0.131	0.085	0.685	0.026
0.227	0.083	0.48	0.219	0.174	0.071	0.198	0.816	0.338	0.198	0.139	0.031	0.63
0.593	0.784	0.147	0.252	0.357	0.327	0.521	0.106	0.246	0.521	0.071	0.066	0.111

CG2932-PA:Bteb2

0.111	0.142	0.044	0.012	0.173	0.044	0.012	0.142	0.095	0.346
0.035	0.027	0.023	0.011	0.66	0.023	0.009	0.027	0.102	0.018
0.664	0.804	0.878	0.937	0.026	0.878	0.916	0.804	0.71	0.608
0.19	0.027	0.055	0.04	0.142	0.055	0.064	0.027	0.093	0.028

CG3065-PB:CG3065

0.111	0.142	0.044	0.012	0.173	0.044	0.016	0.142	0.095	0.12
0.035	0.027	0.023	0.011	0.66	0.023	0.017	0.027	0.102	0.422
0.664	0.804	0.878	0.937	0.026	0.878	0.912	0.804	0.71	0.326
0.19	0.027	0.055	0.04	0.142	0.055	0.055	0.027	0.093	0.132

CG31365-PA:CG31365

0.011	0.189	0.165	0.14	0.705	0.402	0.049	0.074	0.402	0.022	0.242	0.354	0.12
0.012	0.432	0.223	0.392	0.085	0.015	0.154	0.103	0.015	0.015	0.201	0.354	0.422
0.948	0.195	0.408	0.325	0.139	0.338	0.732	0.44	0.338	0.93	0.328	0.146	0.326
0.029	0.185	0.204	0.144	0.071	0.246	0.065	0.384	0.246	0.033	0.228	0.146	0.132

CG32120-PA:Ly

0.011	0.046	0.044	0.261	0.705	0.402	0.116	0.046	0.402	0.148	0.35	0.044	0.12
0.012	0.859	0.023	0.101	0.085	0.015	0.235	0.859	0.015	0.335	0.16	0.023	0.422
0.948	0.047	0.878	0.495	0.139	0.338	0.489	0.047	0.338	0.381	0.064	0.878	0.326
0.029	0.049	0.055	0.143	0.071	0.246	0.16	0.049	0.246	0.137	0.426	0.055	0.132

CG32466-PA:rn

0.074	0.705	0.402	0.14	0.142	0.402	0.14	0.124	0.529	0.123	0.046	0.233	0.346
0.116	0.085	0.015	0.392	0.027	0.015	0.392	0.345	0.165	0.411	0.859	0.141	0.018
0.661	0.139	0.338	0.325	0.804	0.338	0.325	0.174	0.138	0.329	0.047	0.48	0.608
0.149	0.071	0.246	0.144	0.027	0.246	0.144	0.357	0.168	0.138	0.049	0.147	0.028

CG3340-PA:Kr

0.074	0.317	0.129	0.021	0.705	0.402	0.259	0.142	0.044	0.405	0.074	0.202	0.204
0.116	0.379	0.726	0.021	0.085	0.015	0.075	0.027	0.023	0.288	0.103	0.399	0.489
0.661	0.083	0.068	0.899	0.139	0.338	0.553	0.804	0.878	0.133	0.44	0.071	0.122
0.149	0.221	0.077	0.059	0.071	0.246	0.114	0.027	0.055	0.175	0.384	0.327	0.185

CG3851-PA:odd

0.232	0.35	0.402	0.162	0.142	0.044	0.014	0.705	0.095	0.233
0.053	0.16	0.015	0.723	0.027	0.023	0.012	0.085	0.102	0.026
0.364	0.064	0.338	0.032	0.804	0.878	0.915	0.139	0.71	0.63
0.352	0.426	0.246	0.084	0.027	0.055	0.059	0.071	0.093	0.111

CG4427-PA:EP2237

0.111	0.142	0.044	0.012	0.173	0.044	0.012	0.142	0.095	0.12
0.035	0.027	0.023	0.011	0.66	0.023	0.009	0.027	0.102	0.422
0.664	0.804	0.878	0.937	0.026	0.878	0.916	0.804	0.71	0.326
0.19	0.027	0.055	0.04	0.142	0.055	0.064	0.027	0.093	0.132

CG5245-PA:CG5245

0.111	0.073	0.072	0.122	0.142	0.529	0.125	0.142	0.044	0.123	0.062	0.233	0.563
0.035	0.072	0.019	0.433	0.027	0.165	0.405	0.027	0.023	0.411	0.016	0.141	0.146
0.664	0.073	0.017	0.319	0.804	0.138	0.328	0.804	0.878	0.329	0.816	0.48	0.146
0.19	0.783	0.892	0.127	0.027	0.168	0.142	0.027	0.055	0.138	0.106	0.147	0.146

CG5249-PA:CG5249

0.192	0.142	0.402	0.261	0.705	0.233	0.126	0.059	0.072	0.215	0.705	0.402	0.12
0.102	0.027	0.015	0.101	0.085	0.141	0.405	0.049	0.019	0.314	0.085	0.015	0.422
0.411	0.804	0.338	0.495	0.139	0.48	0.331	0.386	0.017	0.219	0.139	0.338	0.326
0.296	0.027	0.246	0.143	0.071	0.147	0.138	0.506	0.892	0.252	0.071	0.246	0.132

CG5557-PA:sqz

0.074	0.705	0.402	0.14	0.142	0.402	0.14	0.124	0.529	0.12
0.116	0.085	0.015	0.392	0.027	0.015	0.392	0.345	0.165	0.422
0.661	0.139	0.338	0.325	0.804	0.338	0.325	0.174	0.138	0.326
0.149	0.071	0.246	0.144	0.027	0.246	0.144	0.357	0.168	0.132

CG5669-PA:CG5669

0.111	0.142	0.044	0.012	0.173	0.044	0.012	0.142	0.095	0.12
0.035	0.027	0.023	0.011	0.66	0.023	0.009	0.027	0.102	0.422
0.664	0.804	0.878	0.937	0.026	0.878	0.916	0.804	0.71	0.326
0.19	0.027	0.055	0.04	0.142	0.055	0.064	0.027	0.093	0.132

CG5683-PA:Aef1

0.524	0.35	0.402	0.164	0.35	0.402	0.164	0.35	0.402	0.164	0.35	0.402	0.12
0.169	0.16	0.015	0.394	0.16	0.015	0.394	0.16	0.015	0.394	0.16	0.015	0.422
0.139	0.064	0.338	0.306	0.064	0.338	0.306	0.064	0.338	0.306	0.064	0.338	0.326
0.169	0.426	0.246	0.136	0.426	0.246	0.136	0.426	0.246	0.136	0.426	0.246	0.132

CG6824-PA:ovo

0.074	0.242	0.402	0.012	0.046	0.129	0.013	0.059	0.072	0.177
0.116	0.201	0.015	0.013	0.859	0.726	0.012	0.049	0.019	0.736
0.661	0.328	0.338	0.943	0.047	0.068	0.947	0.386	0.017	0.012
0.149	0.228	0.246	0.032	0.049	0.077	0.028	0.506	0.892	0.076

CG7204-PA:CG7204

0.01	0.142	0.055	0.405	0.189	0.202	0.125	0.705	0.121	0.12
0.003	0.027	0.127	0.288	0.432	0.399	0.405	0.085	0.121	0.422
0.884	0.804	0.056	0.133	0.195	0.071	0.328	0.139	0.121	0.326
0.104	0.027	0.762	0.175	0.185	0.327	0.142	0.071	0.638	0.132

CG7672-PB:gl

0.111	0.35	0.129	0.148	0.242	0.402	0.066	0.705	0.402	0.259	0.35	0.044	0.12
0.035	0.16	0.726	0.335	0.201	0.015	0.215	0.085	0.015	0.075	0.16	0.023	0.422
0.664	0.064	0.068	0.381	0.328	0.338	0.601	0.139	0.338	0.553	0.064	0.878	0.326
0.19	0.426	0.077	0.137	0.228	0.246	0.118	0.071	0.246	0.114	0.426	0.055	0.132

CG7847-PA:sr

0.011	0.173	0.044	0.039	0.142	0.044	0.012	0.173	0.044	0.012
0.012	0.66	0.023	0.022	0.027	0.023	0.011	0.66	0.023	0.011
0.948	0.026	0.878	0.877	0.804	0.878	0.937	0.026	0.878	0.928
0.029	0.142	0.055	0.061	0.027	0.055	0.04	0.142	0.055	0.049

CG8159-PA:CG8159

0.524	0.073	0.529	0.025	0.189	0.529	0.124	0.173	0.233	0.02	0.242	0.247	0.12
0.169	0.072	0.165	0.013	0.432	0.165	0.416	0.66	0.141	0.013	0.201	0.248	0.422
0.139	0.073	0.138	0.929	0.195	0.138	0.322	0.026	0.48	0.935	0.328	0.258	0.326
0.169	0.783	0.168	0.034	0.185	0.168	0.139	0.142	0.147	0.032	0.228	0.247	0.132

CG9768-PA:hkb

0.111	0.142	0.044	0.012	0.173	0.044	0.25	0.317	0.529	0.357
0.035	0.027	0.023	0.011	0.66	0.023	0.095	0.379	0.165	0.068
0.664	0.804	0.878	0.937	0.026	0.878	0.467	0.083	0.138	0.417
0.19	0.027	0.055	0.04	0.142	0.055	0.188	0.221	0.168	0.158

CG9786-PB:hb

0.074	0.205	0.529	0.115	0.142	0.121	0.023	0.046	0.38	0.666
0.116	0.285	0.165	0.403	0.027	0.466	0.037	0.859	0.15	0.144
0.661	0.084	0.138	0.347	0.804	0.121	0.831	0.047	0.213	0.112
0.149	0.426	0.168	0.136	0.027	0.293	0.109	0.049	0.257	0.079

CG9895-PA:CG9895

0.192	0.142	0.044	0.012	0.173	0.044	0.012	0.142	0.095	0.12
0.102	0.027	0.023	0.011	0.66	0.023	0.009	0.027	0.102	0.422
0.411	0.804	0.878	0.937	0.026	0.878	0.916	0.804	0.71	0.326
0.296	0.027	0.055	0.04	0.142	0.055	0.064	0.027	0.093	0.132

References

Bartsevich VV, Juliano RL (2000) Regulation of the MDR1 gene by transcriptional repressors selected using peptide combinatorial libraries. Mol Pharm 58: 1-10.

Benos PV, Lapedes AS, Stormo GD (2002) Probabilistic Code for DNA Recognition by Proteins of the EGR Family. J Mol Biol 323: 701-727.

Biesiada E, Razandi M, Levin ER (1996) Egr-1 activates basic fibroblast growth factor transcription. Mechanistic implications for astrocyte proliferation. J Biol Chem 271: 18576-18581.

Bulyk ML, Johnson PLF, Church GM (2002) Nucleotides of transcription factor binding sites exert interdependent effects on the binding affinities of transcription factors. Nucleic Acids Res 30: 1255-1261.

Bulyk ML, Huang X, Choo Y, Church GM (2001) Exploring the DNA-binding specificities of zinc fingers with DNA microarrays. Proc Natl Acad Sci USA 98: 7158-7163.

Erno H, Kury P, Botteri FM, Monard D (1996) A Krox binding site regulates protease nexin-1 promoter activity in embryonic heart, cartilage and parts of the nervous system. Mech Dev 60: 139-150.

Kishikawa S, Murata T, Kimura H, Shiota K, Yokoyama KK (2002) Regulation of transcription of the Dnmt1 gene by Sp1 and Sp3 zinc finger proteins. Euro J Biochem 269: 2961-2970.

Koyano-Nakagawa N, Nishida J, Baldwin D, Arai K, Yokota T (1994) Molecular cloning of a novel human cDNA encoding a zinc finger protein that binds to the interleukin-3 promoter. Mol Cell Biol 14: 5099-5107.

Kriwacki RW, Schultz SC, Steitz TA, Caradonna JP (1992) Sequence-specific recognition of DNA by zinc-finger peptides derived from the transcription factor Sp1. Proc Natl Acad Sci USA 89: 9759-9763.

Kyo S, Takakura M, Taira T, Kanaya T, Itoh H, et al. (2000) Sp1 cooperates with c-Myc to activate transcription of the human telomerase reverse transcriptase gene (hTERT). Nucleic Acids Res 28: 669-677.

Lee JS, Ngo H, Kim D, Chung JH (2000) Erythroid Kruppel-like factor is recruited to the CACCC box in the beta-globin promoter but not to the CACCC box in the gamma-globin promoter: the role of the neighboring promoter elements. Proc Natl Acad Sci USA 97: 2468-2473.

Mandel-Gutfreund Y, Baron A, Margalit H (2001) A structure-based approach for prediction of protein binding sites in gene upstream regions. Proc of the Pac Symp Biocomput: 139-150.
Nonchev S, Vesque C, Maconochie M, Seitanidou T, Ariza-McNaughton L, et al. (1996) Segmental expression of Hoxa-2 in the hindbrain is directly regulated by Krox-20. Development 122: 543-554.
Perkins A (1999) Erythroid Kruppel like factor: from fishing expedition to gourmet meal. Intl J Biochem Cell Biol 31: 1175-1192.
Raney AK, McLachlan A (1995) Characterization of the hepatitis B virus large surface antigen promoter Sp1 binding site. Virology 208: 399-404.
Sham MH, Vesque C, Nonchev S, Marshall H, Frain M, et al. (1993) The zinc finger gene Krox20 regulates HoxB2 (Hox2.8) during hindbrain segmentation. Cell 72: 183-196.
Skerka C, Decker EL, Zipfel PF (1995) A regulatory element in the human interleukin 2 gene promoter is a binding site for the zinc finger proteins Sp1 and EGR-1. J Biol Chem 270: 22500-22506.
Teunissen BEJ, Amersfoorth SCMv, Opthof T, Jongsma HJ, Bierhuizen MFA (2002) Sp1 and Sp3 activate the rat connexin40 proximal promoter. Biochem Biophys Res Comm 292: 71-78.
